# How the Dominant Reading Direction Changes Parafoveal Processing: A Combined EEG/Eye‐Tracking Study

**DOI:** 10.1111/psyp.70205

**Published:** 2025-12-04

**Authors:** Xin Huang, Hezul Tin‐Yan Ng, Chien Ho Lin, Ming Yan, Olaf Dimigen, Werner Sommer, Urs Maurer

**Affiliations:** ^1^ School of Psychology Nanjing Normal University Nanjing China; ^2^ Department of Psychology The Chinese University of Hong Kong Hong Kong China; ^3^ Wofoo Joseph Lee Consulting and Counselling Psychology Research Centre Lingnan University Hong Kong China; ^4^ Department of Psychology University of Macau Macau China; ^5^ Experimental Psychology University of Groningen Groningen the Netherlands; ^6^ Department of Psychology Humboldt‐Universität zu Berlin Berlin Germany; ^7^ Department of Physics and Life Science Imaging Center Hong Kong Baptist University Hong Kong China; ^8^ Department of Psychology Zhejiang Normal University Jin Hua China; ^9^ Faculty of Education National University of Malaysia Kuala Lumpur Malaysia; ^10^ Brain and Mind Institute The Chinese University of Hong Kong Hong Kong China

**Keywords:** Chinese, combined EEG/eye tracking, FRPs, vertical reading experience, visual word recognition

## Abstract

Reading directions vary across writing systems. Through long‐term experience, readers adjust their visual systems to the dominant reading direction in their writing systems. However, little is known about the neural correlates underlying these adjustments because different writing systems do not just differ in reading direction, but also in visual and linguistic properties. Here, we took advantage of the fact that Chinese is read to different degrees in left‐to‐right or top‐to‐bottom directions in different regions. We investigated visual word processing in participants from Taiwan (both top‐to‐bottom and left‐to‐right directions) and from mainland China (only left‐to‐right direction). We used combined EEG/eye‐tracking with a saccade‐contingent parafoveal preview manipulation to investigate how the dominant reading direction shapes neural visual processing while participants read 5‐word lists. Fixation‐related potentials (FRPs) showed a reduced late N1 effect (preview positivity), but this effect was modulated by prior experience with a specific reading direction. Results replicated previous findings that valid previews facilitate visual word processing, as indicated by reduced FRP activation. Critically, the results provide the first neuroelectric evidence that this facilitation effect depends on experience with a given reading direction. The findings provide insight into how cultural experience shapes the way people process visual information and demonstrate how a person's everyday visual experience can influence how the brain processes parafoveal information.

## Impact Statement

This study uniquely demonstrates how reading experience shapes neural mechanisms of parafoveal processing. By comparing Taiwanese and Mainland Chinese readers using simultaneous EEG‐eye tracking, we reveal that directional reading experience directly modulates visual word recognition. These findings advance neurocognitive models of reading by showing how cultural practices shape fundamental cognitive processes.

## 1 Introduction

A neurocognitive framework proposing that the brain and mind are shaped by sociocultural experience has gained much attention (Han and Northoff 2008). Writing systems and reading direction, as aspects of sociocultural experience, are considered important in shaping neurocognitive networks (Kazandjian and Chokron 2008). Cultures have developed different writing systems that vary in terms of their reading direction. For example, Roman alphabetic languages are written and read from left to right, whereas Hebrew and Arabic Abjad are read from right to left. The Ancient Greek “boustrophedon” writing style illustrates different reading directions in the same script, as it alternated between left–right and right–left directions, like an ox turns when plowing a field. Although modern Chinese is most frequently written from left to right, it was traditionally written in a top‐down direction, and many Chinese readers are still regularly exposed to the top‐down direction when reading novels or classical texts. Thus, learning to read Chinese also entails some familiarity with reading in the top‐down direction. Interestingly, how the visual system deals with such differences in reading direction, and whether it undergoes general adaptations during the process of learning to read, is largely unexplored.

Reading experience can influence the size of the perceptual span, that is, the area from which readers pick up information during a fixation (McConkie and Rayner 1975; for a review, see Rayner et al. 2009). The perceptual span is asymmetric across writing systems, extending wider in the reading direction than in the opposite direction. For example, skilled readers of English who read from left to right obtain useful information from an area extending 14–15 letter spaces to the right of fixation but only 3–4 letter spaces to the left (McConkie and Rayner 1976; Rayner et al. 1980; Underwood and McConkie 1985). Also in Japanese, where texts are written either in a horizontal or vertical direction, Osaka (1993) found that the perceptual span was asymmetric toward the direction of reading, depending on whether it was vertical or horizontal. When reading Chinese from left to right, the perceptual span extends one character to the left of fixation but two to four characters to the right (Inhoff and Liu 1997; Yan et al. 2015). Therefore, it is established that the reader's perceptual span shows an asymmetrical pattern, extending further in the direction of reading than against it.

Two explanations have been suggested to account for this asymmetric perceptual span in reading. One is hemispheric specialization, which assumes that if the perceptual span is determined primarily by the hemispheric projections, then languages that are written from left to right should all produce a rightward asymmetry, as dominance for language is typically left hemispheric (Almabruk et al. 2011; Ibrahim and Eviatar 2009, 2012). The visual pathway involves distinct hemispheric projections: left visual field information projects to the right hemisphere, whereas right visual field information projects to the left hemisphere. Since language processing is primarily left hemispheric, this arrangement may benefit readers of left to right scripts like English, as text in their right visual field directly reaches the language‐dominant left hemisphere, potentially enhancing reading efficiency (Ibrahim and Eviatar 2012). Reading experience (i.e., scanning habits), which shapes the perceptual span to align with the habitual reading direction, is an alternative explanation. So far, empirical studies have only supported reading experience accounts but have provided no evidence for the hemispheric dominance account. Pollatsek et al. (1981) tested the extent of the perceptual span in native Israeli Hebrew/English bilinguals who read sentences while a gaze‐contingent moving window extended either up to 14 characters to the left or to the right of fixation. Results showed that reading performance for Hebrew was superior when the asymmetric window was larger to the left, whereas performance for English was superior when the window was larger to the right. In addition, Jordan et al. (2013) provided evidence that the central perceptual span (an area extending 2.5 degrees to either side of fixation; 2.5 degrees encompassed around 9 letters) was skewed according to the overall reading direction. In this study, skilled Arabic readers who were bilingual in Arabic and English read both Arabic and English sentences. In a symmetric window condition, the moving window of normal text extended 0.5 degrees to the left and right of fixation. In an asymmetric condition, the window was increased to 1.5 degrees or 2.5 degrees to either the left or the right. When reading English, performance across window conditions was superior when the window extended rightward. Conversely, when reading Arabic, performance was superior when the window extended leftward. Similar results were replicated for Urdu‐English bilingual readers (Paterson et al. 2014) reading in another left‐running Arabic‐based script. In a later study testing another right to left script, Zhou et al. (2021) determined the perceptual span in Uyghur to cover 5 previous letters to the right of a fixation, and 12 upcoming letters to the left. Culturally acquired directional scanning habits even extend to non‐text reading, namely, picture naming and recall (Padakannaya et al. 2002). On the basis of these results, it is believed that experience with reading in a specific direction can modify the asymmetry of the perceptual span.

Besides the asymmetric perceptual span, reading experience can also influence the preferred viewing locations (PVL) within words (Rayner 1979). Yan et al. (2014) found that in Uighur scripts with a right‐to‐left reading direction, readers showed a rightward‐shifted PVL, meaning that more visual information about the fixated word was projected into the readers' left visual field. In contrast, in scripts that are written from left to right (e.g., English), the PVL is shifted to the left (Deutsch and Rayner 1999).

Whereas most studies that support the reading direction account used horizontal texts to investigate the asymmetry of the perceptual span, the horizontal–vertical contrast may be a better condition to test this account. If readers are used to reading in a vertical direction, they should show a larger perceptual span in vertical texts than readers who are used to reading horizontal texts. When comparing the perceptual span in different writing directions, most previous studies used different scripts for different reading directions (except for studies on Japanese, Mongolian, and traditional Chinese, Naoyuki Osaka and Oda 1991; Su et al. 2020; Yan et al. 2024). Therefore, the observed differences in the asymmetry of perceptual span in different reading directions are confounded with differences in the script systems. A systematic comparison between horizontal and vertical reading of traditional Chinese sentences was initiated by Yan et al. (2019), who aimed to tease apart the effects of language proficiency at a literacy level and direction‐specific reading experience at a perceptual level by using a within‐subject and within‐item design. They reported largely equivalent reading and oculomotor activity in both reading directions among skilled readers from Taiwan. Importantly, during vertical reading, compared to horizontal reading, readers made fixations that were closer to the word center but maintained each fixation for a longer duration. Yan et al. (2024) further examined the horizontal and vertical perceptual spans of traditional Chinese readers from Hong Kong, who were also relatively accustomed to both reading directions. The study revealed that the perceptual span for vertical reading was generally smaller than for horizontal reading, because of participants' lower average familiarity with vertical text. Additionally, the vertical perceptual span increased with the readers' familiarity with vertical text, with those highly familiar with vertical text extending their perceptual span beyond four upcoming characters. These studies provided compelling evidence that readers' perceptual experience in a specific text direction, independent of their language skills, shapes their visual processing of the text. However, since these studies recorded only eye movement data, the differences in neurological activity between the two reading directions remain to be explored.

Building upon these findings, our study aims to further investigate the influence of reading direction on the asymmetry of the perceptual span while controlling for script‐related confounds. By using the Chinese script and having the same participants read the same Chinese stimuli in different directions, we can directly examine how reading experience shapes preview effects. This approach allows us to address a fundamental question in reading research: whether perceptual span asymmetries are primarily shaped by a habitual reading direction or are inherent properties of the writing system. Previous studies could not definitively answer this question because of confounds between script properties and reading direction.

Historically, Chinese sentences were written vertically in columns going from top to bottom, with each new column starting to the left of the preceding one. Only rather recently was the horizontal alignment with a rightward reading direction adopted. The People's Republic of China adopted horizontal alignment in 1956 along with the simplified Chinese orthographic reform, although vertical alignment is still occasionally used (e.g., in older Chinese books). Although the horizontal alignment has been adopted in math and science texts, vertical alignment is still common in novels, newspapers, and magazines in other Chinese‐speaking countries like Taiwan, Hong Kong, and Macau, where traditional Chinese characters are also employed. As a result, both horizontal and vertical reading directions are familiar and efficient to readers of traditional Chinese. In contrast, readers of simplified Chinese (as used in mainland China) are more familiar with horizontal alignments. This provides an opportunity to investigate the effects of experience with different reading directions in the same language.

Previous eye‐tracking studies on traditional Chinese in Taiwan showed findings similar to those on simplified Chinese in mainland China, even though their reading and writing directions are different. For example, phonological (Tsai et al. 2004), morphological (Yen et al. 2008), and semantic information (Tsai et al. 2012) could be accessed in the parafoveal area during horizontal reading, which is consistent with findings on simplified Chinese in mainland China (Liu et al. 2002; Yan et al. 2009; Yan, Zhou, et al. 2012). Even in vertical reading, semantic information was still accessible parafoveally (Pan et al. 2022). However, given that these studies were limited to Taiwanese participants, a direct comparison with oculomotor behavior in mainland Chinese readers is still lacking.

Although there is evidence from eye‐tracking studies that cultural experience influences how readers process information in different reading directions, the neural correlates of these processes are not yet known because of the limitations of standard neuroimaging methods. Event‐related potentials (ERPs) have high temporal resolution, but the pervasive eye movement artifacts and other problems, such as the overlap between the ERP components elicited by successive fixations, have hindered natural reading studies for a long time. However, advances in ocular correction techniques that make use of the eye‐tracking information allow researchers to deal with these eye movement artifacts in EEG (Dimigen 2020). The development of the EEG/eye‐tracking co‐registration technique allows studying unconstrained viewing situations, including natural reading. The technique gathers complementary data about time and space by recording eye movements and brain activity (EEG); that is, eye‐tracking shows where people are looking, whereas EEG reveals when and how the brain reacts to the visual input. By time‐locking the EEG to fixation onsets, we can collect fixation‐related potentials (FRPs) that can capture perceptual and cognitive processes related to the object in the current fixation. In order to investigate reading, the EEG/eye‐tracking co‐registration technique can be combined with traditional eye gaze‐contingent paradigms, including the boundary paradigm (Rayner 1975). In this paradigm, an invisible boundary is embedded in the text. Prior to crossing the boundary, a parafoveal preview stimulus is shown instead of the actual target word. Only when the reader's gaze crosses the boundary is the preview stimulus replaced by the target word. By manipulating the relationship between the preview stimulus and the target word, it is possible to study the types of information readers extract from the parafovea. The perceptual span in reading has traditionally been measured using the moving window paradigm (for review, see Rayner 2014). However, in reading research, particularly in studies combining EEG and eye‐tracking, the boundary paradigm has become the preferred choice (e.g., Dimigen et al. 2012; Degno et al. 2019b; Li et al. 2024), because of several advantages. First, the boundary paradigm provides a more naturalistic reading experience compared to the moving window paradigm. Second, it involves only one display change per trial rather than continuous screen refreshes, resulting in fewer potential visual artifacts in the EEG signal. Third, the boundary paradigm offers precisely controlled experimental conditions with carefully selected word properties, whereas the moving window paradigm aggregates data from diverse fixations across a variety of word types (including both function and content words), resulting in measurements that are primarily differentiated by window size rather than specific lexical characteristics. Although traditionally used for studying preview benefits, the boundary paradigm can also assess perceptual span modulation: a larger identity preview effect suggests more parafoveal information acquisition during previous fixations, indicating a larger perceptual span. For instance, Inhoff et al. (1989) found larger preview effects with normal text compared to letter‐transformed text, indicating that readers obtain more parafoveal information from normal words. Similarly, a reduction of the preview effect has been found when pre‐target words were infrequent (Henderson and Ferreira 1990). In Chinese reading, Yan et al. (2010) reported a larger preview effect from the second post‐boundary word (i.e., word *N* + 2), when word *N* + 1 was more frequent. More recently, Yan and Sommer (2019) demonstrated that emotionally negative foveal words bind more attention than neutral and positive words, leading to a reduced *N* + 2 preview effect.

Studies combining EEG and eye‐tracking found a reduced negativity (termed “preview positivity”) in FRPs following valid as compared to invalid previews in a time window between 200 and 280 ms after fixating the target word *N* + 1 (e.g., Dimigen et al. 2012; Kornrumpf et al. 2016), which was maximal over the occipito‐temporal scalp. Although this effect has often been referred to as “the late N1 effect”, its time window and scalp distribution are similar to the late N1 or the N1 offset (e.g., Maurer et al. 2024; Wang and Maurer 2017, 2020) or N250 component observed in masked priming studies (e.g., Holcomb and Grainger 2007; Huang et al. 2025). Therefore, the neural mechanism may be interpreted as a facilitatory effect of repetition suppression (Dimigen et al. 2012). The time course and scalp distribution (largest over left occipito‐temporal regions) of the effect fit with previous late N1 or N250 findings (e.g., Bentin et al. 1999; Maurer et al. 2005), which have been linked to orthographic processing at the interface between sub‐lexical and whole‐word representations. Previous findings demonstrate that preview effects increase in magnitude when previews and targets share a higher degree of orthographic overlap. The preview positivity is not only observed in word list reading (Dimigen et al. 2012; Niefind and Dimigen 2016), but also in natural sentence reading (Degno et al. 2019a, 2019b; Dimigen and Ehinger 2021; Li et al. 2024).

In contrast to the late N1 component, the early parts of the “N1 effect” have been less frequently reported or investigated. The early N1 effect, which also refers to the N1 onset in some studies (e.g., Maurer et al. 2024; Wang and Maurer 2017, 2020), has been found in visual word processing with unrelated stimuli eliciting larger negativities compared to repeated stimuli (Degno et al. 2019a; Dimigen et al. 2012; Kornrumpf et al. 2016; Li et al. 2015; Niefind and Dimigen 2016). Similar to the late N1 effect, the early N1 effect shows the largest activity in occipito‐temporal regions of the scalp. Compared to the preview positivity, the early N1 effects are usually smaller, less robust, and less consistent. Also, there appears to be a tendency for the early N1 preview effects to be larger in Chinese (Li et al. 2015, 2022, 2024) than in alphabetic languages (i.e., Dimigen and Ehinger 2021), possibly because of the higher visual complexity of Chinese script and its higher demands on visual processing (McBride‐Chang et al. 2011; Zhao et al. 2014). The early N1 has been demonstrated to reflect dual functions in word processing. Primarily, within alphabetic writing systems, it is sensitive to the systematic mapping of visual features onto position‐specific letter representations (see Grainger and Holcomb 2010, for a review). Additionally, it is a neural indicator of expertise with the script system, as evidenced by enhanced amplitudes in response to letter strings from familiar orthographic systems relative to both visual control stimuli and unfamiliar writing systems (Maurer et al. 2005, 2008). Contemporary investigations employing rigorously controlled stimuli have further elucidated the temporal dynamics of this print tuning effect. These studies indicate that print‐specific processing predominantly manifests in the early N1 component, suggesting an earlier emergence of specialized orthographic processing than previously understood (Eberhard‐Moscicka et al. 2016; Wang and Maurer 2017, 2020).

Using the combined EEG and eye‐tracking technique, Simola et al. (2009) investigated how hemispheric differences and reading direction influence parafoveal processing. When presenting words to either the left visual field (LVF) or right visual field (RVF), they found a parafoveal‐on‐foveal effect in the P2 component (as known as preview positivity) that distinguished between words and nonwords, but only for RVF presentations. They attributed this effect to attention being oriented according to habitual reading direction, suggesting that long‐term reading experience in a particular direction creates visual field asymmetries in parafoveal‐on‐foveal effects. However, their study design could not fully separate the contributions of three key factors: structural brain organization (i.e., left hemisphere language dominance), attentional deployment, and perceptual expertise. Specifically, when words appeared in the RVF, both attention and left hemisphere language processing were simultaneously engaged, making it difficult to determine their independent contributions. This limitation highlights the need for future studies that can effectively dissociate these factors.

The present study co‐registered EEG and eye movements in the boundary paradigm to investigate the neural correlates underlying the preview effects in two participant groups that differ with regard to their experience with different reading directions, but essentially use the same script system. To this end, we recruited participants from mainland China, where Chinese is written from left to right, and participants from Taiwan, where Chinese is written in both top‐down and left‐to‐right directions but more often top‐down. Both groups were tested with the same materials in both vertical and horizontal directions. Importantly, only characters that are identical in simplified and traditional Chinese script were used as materials.

We are interested in the early and late N1 effects, which allow us to distinguish temporal subcomponents within the N1 time range. For the late part of the N1 component, we expected reduced (less negative) amplitudes after identical previews as compared to unrelated previews, whereas for the early N1, we expected increased (more negative) amplitudes after identical previews as compared to unrelated previews. The early N1 is thought to reflect the initial phase of sublexical orthographic processing and to be sensitive to elemental features of the stimulus, whereas the late N1 is thought to reflect the partial, trans‐saccadic repetition priming that activates abstract orthographic and phonological representations. On the basis of previous literature (e.g., Dimigen et al. 2012; Huang et al. 2025), for both components, we expected to observe preview‐related amplitude modulations in both time windows. This effect was expected to be similar for the two groups in the horizontal reading direction, as Taiwanese readers are also exposed to horizontal alignment of texts, for example, on digital devices (see the results of a self‐report questionnaire below); however, because of more extensive practice of Taiwanese readers, their N1 reduction in the top‐down direction should be larger than for mainland Chinese readers. Regarding the direction differences, we hypothesized that Taiwanese participants would show larger preview effects in the vertical direction than in the horizontal direction, whereas Mainlanders would show larger late N1 amplitude reduction in the horizontal direction than in the vertical direction. In addition, for eye movement measures, we expected a preview effect, with fixations after identical previews being shorter than those after unrelated previews. Specifically, we expected that the size of the preview benefit would depend on, both, participant group and reading direction in a three‐way interaction: The preview effect was expected to be similar for the two groups in the left–right reading direction, but larger for the Taiwanese than the mainland Chinese group in the top‐down direction because of the presumably larger downward perceptual span of the Taiwanese group.

All methods and proposed analyses for the experiment were preregistered at https://osf.io/34u92/.

## 2 Methods

### 2.1 Participants

Thirty native Chinese (Mandarin) speakers, originally from mainland China (16 females; mean age = 20.5 years, SD = 2.56), and another 30 native Chinese (Mandarin) speakers, originally from Taiwan (16 females; mean age = 22 years, SD = 2.87), participated in the combined EEG/eye‐tracking experiment. All participants were college students studying in Hong Kong. At the time they were recruited, participants had resided in Hong Kong for 2 years on average; the two groups did not differ in the time they had lived in Hong Kong (Mainlanders: *M* = 1.94 years, SD = 2.11, range: 0.17–8 years; Taiwanese: *M* = 2.07, SD = 1.60, range: 0.08–6 years). Importantly, before coming to Hong Kong, members of both groups had continuously lived in their respective home regions.

A self‐report questionnaire was administered to evaluate participants' reading and writing experiences in horizontal and vertical directions before and after moving to Hong Kong. Participants reported their exposure to vertically and horizontally aligned texts across 10 different media types: magazines, books, comics, newspapers, textbooks, smart device content, road signs, billboards, slogans/leaflets, and advertisements (using a 0–4 scale, where 0 = never and 4 = always). Reading time for different text categories was also evaluated. Additionally, participants reported their age of first exposure to horizontal and vertical text directions, as well as their writing experiences in both directions.

Repeated measures ANOVAs revealed a significant main effect of Direction, a significant main effect of Group, and a significant Direction × Group interaction on the average of general and textbook reading (*F*s > 4.59, *p*s < 0.036). Post hoc t‐tests showed that Mainlanders had significantly more horizontal than vertical experience (*t*_(58)_ = 13.00, *p* < 0.001), whereas Taiwanese showed no significant directional difference (*t*_(58)_ = 1.25, *p* = 0.60). Comparing between groups for each direction, Taiwanese had more vertical experience than Mainlanders (*t*_(58)_ = −1.55, *p* < 0.001), whereas Mainlanders had more horizontal experience than Taiwanese (*t*_(58)_ = 5.89, *p* < 0.001). Similar findings were obtained after participants moved to Hong Kong, although the repeated measures ANOVAs revealed no significant main effect of Group (*F*_(1,58)_ = 0.08, *p* = 0.78; refer to the Supporting Information for more details).

Hence, as shown in our questionnaire data, Mainland readers showed dominant exposure to and expertise with horizontal reading, while also having some experience with vertical text. On the other hand, Taiwanese readers are regularly exposed to both horizontal and vertical text formats in their daily lives, particularly among younger generations who frequently use electronic devices. This extensive exposure potentially provides them with enhanced proficiency in horizontal reading (Sun 2010), suggesting a comparable proficiency across different text orientations. When comparing the two groups, Taiwanese readers reported more extensive experience with vertical text than Mainland readers, whereas Mainland readers reported more experience with horizontal text than Taiwanese readers. This pattern suggests that although both groups have exposure to both reading directions, their relative expertise may differ on the basis of the predominant reading direction in their respective environments. All participants were right‐handed, without dyslexia or ADHD (self‐report), and showed normal or corrected‐to‐normal vision (as assessed before the experiment with the Freiburg Visual Acuity and Contrast Test; Bach 1996). Written informed consent was obtained prior to the experiment. All participants were reimbursed with 50 Hong Kong dollars (about 7 USD) per hour. The study was approved by the Joint Chinese University of Hong Kong–New Territories East Cluster Clinical Research Ethics Committee.

### 2.2 Materials

Two‐character words were selected that occur in both traditional and simplified Chinese with the same meaning in Taiwan and mainland China; hence, the visual forms of these words are identical in both regions. Words that were region‐specific or represented names were excluded. Only medium‐ or high‐frequency words in both regions were selected (mainland Chinese, WF‐MC: *M* = 2.26, SD = 0.47, range: 1.51–3.57, retrieved from the SUBTLEX‐CH corpus; Cai and Brysbaert 2010; Taiwanese Chinese, WF‐TC: *M* = 1.55, SD = 0.63, range: 0–3.21,^1^ retrieved from the Sinica Corpus, Chen et al. 1996).

For the parafoveal preview manipulation at the target word position, 72 critical words were selected. These words were presented twice as post‐boundary target words (once after an identical and once after an unrelated preview) and twice as parafoveal previews (once as an identical preview and once as an unrelated preview). To counterbalance the two reading directions and the assignment of items to a particular direction, we created two sets of words by matching the number of strokes and word frequencies in mainland Chinese and Taiwanese.

### 2.3 Construction of Word Lists

Target words and their previews were embedded within lists of other nouns (“fillers”). Each list consisted of five words. Specifically, to create the 5‐word lists, we selected 576 filler words (72 lists × 4 words × 2 sets), which were also presented twice during the experiment. Filler words were matched with target words regarding word frequency (according to the Sinica database and the SUBTLEX‐CH database) and the number of strokes per character in the first and second positions. The pre‐target words were of medium to high frequency (WF‐MC > 2.27, WF‐TC ≥ 0.47) and of low to medium visual complexity (stroke number < 21).

In total, we created 288 (144 × 2 directions) lists consisting of one critical word and four filler words each. Words in a list were phonologically and semantically unrelated and did not orthographically overlap (no homophones, shared semantic or phonetic radicals, see below for details). The target words were placed either at list positions two, three, or four; accordingly, the pre‐target words were placed at list positions one, two or three. In order to avoid visual overlap between the preview and the post‐boundary target word at the critical list position, the target word was always presented in a different font compared to the parafoveal preview word (this is typical for masked priming studies to reduce the visual overlap between primes and targets, see Holcomb and Grainger 2006). In addition to the fonts of pre‐target and target words, we also manipulated the fonts for other words in the lists, consistently introducing font changes between the previewed and the currently fixated words. This implies that the fonts of previews and pre‐target words were the same. If the preceding words were presented in a Kaiti font, the following words were presented in PMingLiu font, and vice versa. The other filler words following each other were presented either in the same font (50%) or in the other font (50%), precluding the usefulness of font type as a cue for the upcoming target words.

#### 2.3.1 Preview‐Target Pairs

As a basis for constructing the critical target nouns and their respective identical or unrelated parafoveal previews, we took a set of 72 pairs of Chinese two‐character nouns for each reading direction (e.g., 巨星–巨星 and 巴掌–巴掌), yielding the basis for identical previews. For these identical noun pairs, 72 unrelated noun pairs were created by exchanging the preview word with a word of a similar number of strokes and frequency, yielding two new pairs without any semantic or other associations (e.g., 字典–巨星and 池塘–巴掌). In the following, such a set of an identical word pair and its unrelated recombination is called a “preview‐target unit”.

#### 2.3.2 Animal Lists

As animal name target words for the reading task (animal name detection), we created an additional 30 lists (15 × 2 sets), which contained the name of an animal equiprobably at one of the five list positions (cf. Dimigen et al. 2012). This task was used to keep participants engaged during the experiment. The embedded animal names had a mean number of 19.53 strokes (SD = 6.73), a mean frequency of 1.91 (SD = 0.43) in simplified Chinese and 1.50 in traditional Chinese (SD = 0.39) respectively, and were matched with the filler words in the animal lists in terms of stroke number and frequency (*t*s < 1.16, *p*s > 0.26). Except for the embedded animal names, the word lists containing an animal name were indistinguishable from the lists used in regular trials. They followed the same design principles, containing the same preview manipulations (animals only in target but not in preview positions in unrelated preview trials) in the same proportions as regular trials. During the experiment, the 15 animal lists were presented randomly among the 72 target lists of each direction. Data from the animal lists were excluded from analyses.

#### 2.3.3 Balancing

Lists were constructed with the aim of minimizing the orthographic, phonological, and semantic overlap between fillers and the embedded words of the preview‐target unit. Additionally, there were no characters sharing similar pronunciations within a given word list. Stroke numbers and word frequencies were matched between filler and target words (see Table 1). Word selection was constrained by traditional‐simplified character differences, the need for phonologically and semantically unrelated nouns without orthographic overlap, allowing only word frequency and stroke number to be controlled while maintaining an adequate stimulus pool. Twenty‐four additional participants (12 each from Taiwan and mainland China; 10 participants per group completed ratings post‐study, per reviewer request) who did not participate in the experiment rated the semantic relatedness of each word list on a scale from 1 to 5. With a mean score of 1.59 (SE = 0.03) and 1.60 (SE = 0.02) in each set, no significant difference was found for semantic relatedness (*F*s < 1.38, *p*s > 0.24). For each direction, lists were presented randomly intermixed.

**TABLE 1:** Similarity measures for targets and fillers in the two sets of stimuli.

Measure	Target	Filler	*p*	
Strokes in Set 1	18.31 (3.57)	17.73 (3.11)	0.30	*n.s*.
Word Frequency (SUBTLEX‐CH) in Set 1	2.28 (0.39)	2.23 (0.24)	0.43	*n.s*.
Word Frequency (Sinica) in Set 1	0.002 (0.002)	0.002 (0.001)	0.22	*n.s*.
Strokes in Set 2	18.83 (3.01)	18.21 (3.11)	0.23	*n.s*.
Word Frequency (SUBTLEX‐CH) in Set 2	2.25 (0.40)	2.28 (0.27)	0.60	*n.s*.
Word Frequency (Sinica) in Set 2	0.002 (0.002)	0.002 (0.002)	0.26	*n.s*.

*Note:* Given are means across words. Standard deviations are provided in parentheses.

For both reading directions, the target words were matched according to the number of strokes and word frequency in mainland Chinese and Taiwanese Mandarin. To equate the two sets of stimuli, the fillers in the two stimulus sets were also matched (see Table 2). Furthermore, the materials used in the two sets were counterbalanced across participants.

**TABLE 2:** Similarity measures for fillers in the two sets of stimuli.

Measure	Set 1	Set 2	*p*	
Target: Strokes	18.32 (3.57)	18.83 (3.01)	0.35	*n.s*.
Target: Word Frequency (SUBTLEX‐CH)	2.28 (0.39)	2.25 (0.40)	0.67	*n.s*.
Target: Word Frequency (Sinica)	0.002 (0.002)	0.002 (0.001)	0.49	*n.s*.
Filler: Strokes	17.73 (3.11)	18.21 (3.11)	0.36	*n.s*.
Filler: Word Frequency (SUBTLEX‐CH)	2.24 (0.24)	2.28 (0.27)	0.29	*n.s*.
Filler: Word Frequency (Sinica)	0.002 (0.002)	0.002 (0.002)	0.28	*n.s*.

*Note:* Given are means across words. All standard deviations are provided in parentheses.

### 2.4 Procedure

Participants were seated in a dimly lit electrically shielded chamber at a distance of 90 cm from a monitor (24 in. BenQ ZOWIE XL2411K, resolution: 1920 × 1080 pixels; vertical refresh rate: 144 Hz). In two separate blocks, participants read the word lists horizontally or vertically, with a short pause within each block. The order of presentation (vertical reading first or horizontal reading first) was counterbalanced across the participants. The words in the two sets were also counterbalanced across the vertical and horizontal conditions. During the experiment, two identical monitors were used; one was oriented horizontally and one vertically for the horizontal and vertical reading directions, respectively. Participants switched between these monitors between blocks. During a given reading direction block, the appropriate monitor was used, while the other monitor was moved aside. Participants were instructed to read each list and to indicate at the end whether it had contained an animal name.

**FIGURE 1:**
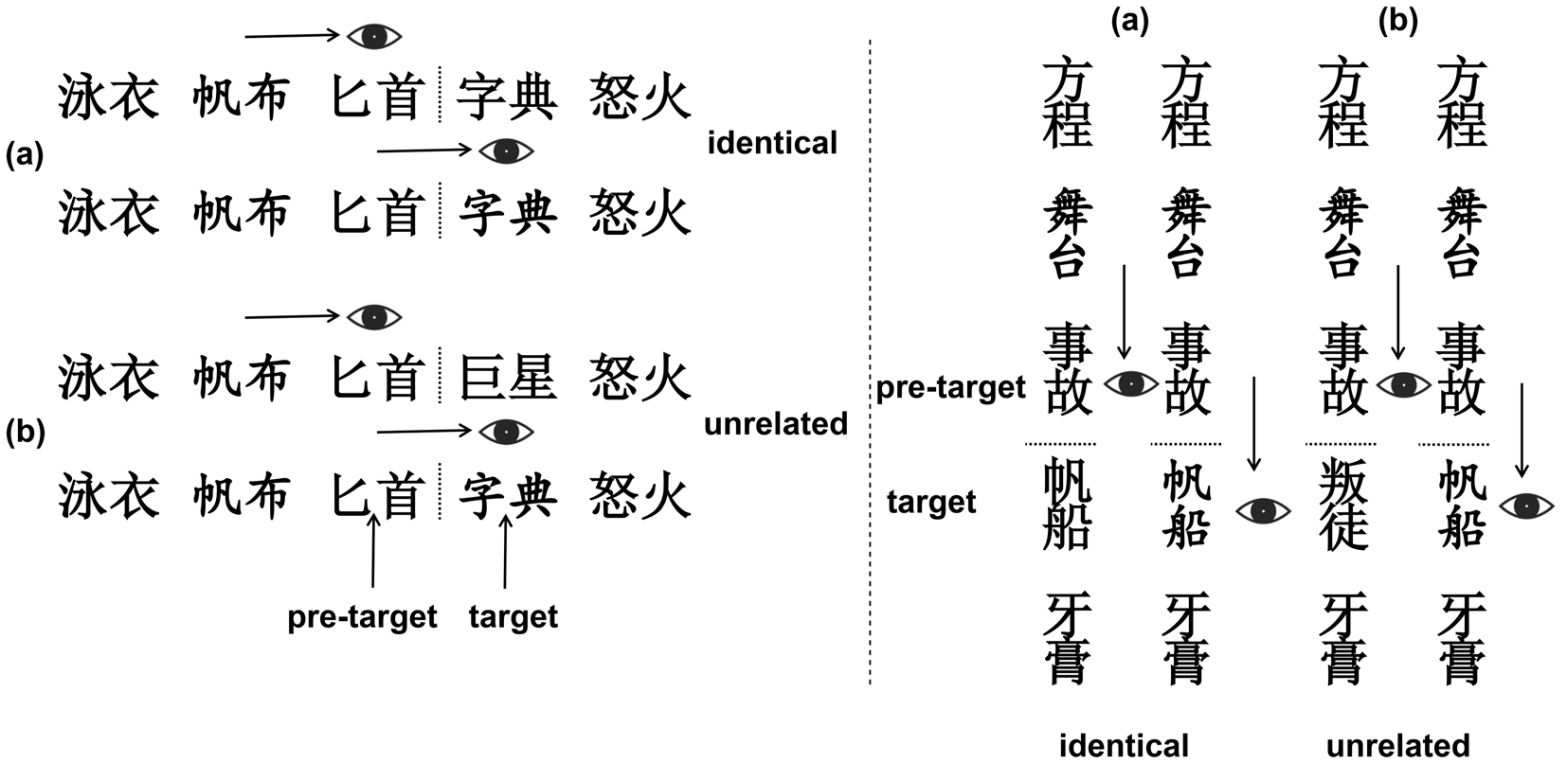
Illustration of experimental conditions. Participants read 5‐word lists with the task of detecting an occasional animal name in the lists. For one word in each list, the parafoveal preview was manipulated using the gaze‐contingent boundary paradigm. Although participants' eyes were still looking at the pre‐target word, the parafoveal preview word could be either (a) identical or (b) unrelated to the target word fixated after the saccade. Left panel: Horizontal reading direction. Right panel: Vertical reading direction.

The trial example is illustrated in Figure 1. Horizontal and vertical trials began with the presentation of a fixation cross on the left or top of the screen, respectively. After a fixation on this point was registered by the eye‐tracker, the list of five words appeared on the horizontal or vertical midline, respectively. Words were presented in black on a white background. Each two‐character word in the list extended to a visual angle of 1.8° horizontally or vertically, depending on the reading direction. In addition, there was one empty character space between the words. The visual angle between the right/lower edge of the pre‐target word and the left/upper edge of the target word was 5.5°,^2^ and an invisible boundary was placed at 2.5° between words. In this case, the inner edge of the preview was located at approximately 3.7 degrees, falling within the parafoveal region (< 5 degrees), whereas the outer edge extended to around 7.3 degrees, considered the peripheral visual field. Therefore, a significant portion of the preview information, especially the character closer to the fixation point, was still within the parafoveal area. Importantly, in conventional Chinese script, characters or words are not separated by spaces (e.g., Hoosain 1992). Here, spaces were inserted between words in order to (1) ensure that the previews were positioned within the extrafoveal vision rather than at the edge of foveal vision, (2) decrease the probability of incorrectly attributing fixations to words due to errors in eye‐tracking, and (3) reduce the chance of late saccade‐contingent changes in the display. Because of these reasons, space insertions have been used in other eye‐tracking studies as well (e.g., Li et al. 2024).

As shown in Figure 1, following the list onset, participants read the five words, moving their eyes freely over the text. After finishing reading, they looked at the final fixation point. After 500 ms, a blank screen appeared, and participants used two buttons to respond with their left or right index fingers whether or not they had seen an animal name in the list. The assignment of yes or no responses to the left or right index finger was counterbalanced across participants. Participants read six lists for practice.

Display change awareness was assessed after the experiment. Participants were first asked whether they had noticed “anything strange about the visual display of the text” (White et al. 2005). They were asked again if they had noticed any changes if they answered “no,” after which they were informed that changes had occurred. If the answer was “yes,” participants were asked to (1) estimate the number of changes perceived, (2) report the identity of some of the preview words, and (3) report the list positions at which changes had occurred (see Dimigen et al. 2012).

### 2.5 EEG Recording

The EEG was recorded from 64 Ag/AgCl scalp electrodes mounted in a textile cap at standard 10–10 system positions and referenced online against the CPz electrode. Two electro‐oculogram (EOG) electrodes were placed on the outer canthus of each eye, and one EOG electrode was placed on the infraorbital ridge of the left eye. Signals were amplified with an EEGO amplifier system (Advanced Neuro Technology, Enschede, Netherlands) at a band‐pass of 0.01–70 Hz and sampled at 1000 Hz. Impedances were kept below 20 kΩ.

### 2.6 Eye Movement Recording

Eye movements were recorded binocularly at a sampling rate of 1000 Hz using an Eyelink 1000 plus eye tracking system (SR Research) in the desktop‐mounted (remote) configuration. Head position was stabilized via the chin rest of the tracker. A 9‐point calibration was completed at the beginning of the experiment and before each change in reading direction. Extra calibrations were performed whenever a check failed. Calibration was accepted when the average error was < 0.5° and the maximum error < 0.99°. Furthermore, a 1‐point drift correction check was performed at the beginning of each trial.

### 2.7 Co‐Registration of Eye Movements and EEG

The co‐registration of eye movements and EEG was achieved by sending shared trigger pulses from the presentation PC (running Presentation, Neurobehavioral Systems Inc., Albany, CA) to the EEG and eye tracking computer on each trial through the parallel port. This allowed for accurate offline synchronization of eye movements and EEG signals via the EYE–EEG extension for EEGLAB (http://www.eyetracking‐eeg.org, Dimigen et al. 2011). After synchronization, the temporal offset between the shared markers in both recordings rarely exceeded 1 ms.

### 2.8 Preprocessing of Eye Movement Data

Three eye movement measures were used for data analysis, including first‐fixation durations (FFD), single fixation durations (SFD), and gaze durations (GD). Fixations were determined by Data Viewer software (SR Research). Only fixations that occurred during the first‐pass reading in trials with a correct answer to the animal question were analyzed. This selection ensures that the same trials contribute to both eye‐tracking and FRP analyses. In ERP/FRP analysis, trials with incorrect responses were usually excluded because they may contain error‐related negativity. Specifically, fixations on the area of interest were excluded when the display change occurred too early or too late (i.e., when the display change took more than 10 ms before/after fixation onset on the target character). We also removed trials with FFD < 60 ms or > 600 ms and GD > 800 ms (total number of excluded fixations: 644). Additionally, we excluded fixations on target words in which participants blinked. We removed all trials with an incorrect manual response to the animal question. Taken together, from all participants, we collected 15,724 observations. The trials left for each condition are listed in Table 3.^3^

**TABLE 3:** Mean number of analyzed trials, standard deviations, and range.

	Condition	*M*	SD	Range
Mainlanders	H‐identical	64.43	5.90	50–72
	H‐unrelated	66.07	4.68	57–72
	V‐identical	64.30	6.89	37–72
	V‐unrelated	64.60	6.28	41–72
Taiwanese	H‐identical	62.43	8.27	30–70
	H‐unrelated	62.73	7.09	44–71
	V‐identical	61.43	9.06	39–72
	V‐unrelated	61.80	6.89	37–72

Abbreviations: H, horizontal reading direction; V, vertical reading direction.

### 2.9 EEG Preprocessing

Offline, EEG data were digitally band‐pass filtered using Finite Impulse Response (FIR) with the EEGLAB 2020.0 (Delorme and Makeig 2004) toolbox for MATLAB (version 2018b), between 0.1 Hz and 30 Hz (−6 dB/octave), and re‐calculated to the average reference (Lehmann and Skrandies 1980). Independent component analysis (ICA) was used for ocular correction using procedures implemented in the EYE‐EEG extension. Specifically, following the ICA decomposition, we removed all independent components that showed much more activity during saccades than during fixation periods (saccade/fixation variance ratio > 1.1) following the procedures and threshold recommendations provided in Plöchl et al. (2012) and Dimigen (2020).

After ocular correction, the EEG signal was segmented from 300 ms before to 700 ms after the first fixation onset on a word. The baseline was corrected by subtracting the 150 ms preceding the fixation onset on the target word. Epochs with amplitudes exceeding ±100 μV in any channel (except the EOG) were automatically rejected from further analyses. FRPs were then averaged within and across participants.

After eye movement and FRP preprocessing, across all 60 participants, our screening left us with a total number of 7348 good epochs for the target character in the vertical reading condition and 5741 good epochs in the horizontal reading condition (the trials left for each condition are listed in Table 4). Within each reading direction, there was a similar number of remaining epochs for the unrelated and identical previews (unrelated, *M* = 48.47, SE = 1.03; identical, *M* = 48.31, SE = 1.08). However, the number of remaining trials was significantly different for the two reading directions (main effect, *t*_(59)_ = −13.38, *p* < 0.001) because participants failed the trial‐initial fixation check more often in the horizontal than in the vertical direction. The analysis of variance (ANOVA, with Bonferroni correction on post hoc tests) on the number of remaining trials in the two groups and directions showed that neither the main effect of Group nor the interaction with trial number was significant (*F*s < 1.58, *p*s > 0.21).

**TABLE 4:** Mean number of analyzed trials, standard deviations, and range in EEG data.

	Condition	M	SD	Range
Mainlanders	H‐identical	41.73	7.71	22–57
	H‐unrelated	41.17	7.52	25–57
	V‐identical	56.10	10.91	21–67
	V‐unrelated	57.47	10.21	29–70
Taiwanese	H‐identical	39.87	7.91	20–57
	H‐unrelated	39.93	8.00	25–57
	V‐identical	55.53	11.83	15–69
	V‐unrelated	55.30	10.75	17–70

Abbreviations: H, horizontal reading direction; V, vertical reading direction.

### 2.10 Data Analysis

#### 2.10.1 Eye Movements

Eye movement data were analyzed with linear mixed‐effects models (LMMs) within the *R* environment for statistical computing (R Core Team 2023). We estimated variance components for subjects and for items (i.e., varying intercepts and slopes) using the “lmer” function of the *lme4* package (Bates et al. 2015; version 1.1.27.1) on log‐transformed FFDs, SFDs, and GDs. The within‐subject factors of *Preview* (identical vs. unrelated) and *Direction* (vertical vs. horizontal) and the between‐subject factor *Group* (Taiwanese vs. Mainlander) were coded as fixed factors. For the Preview factor, we used a treatment contrast approach, where identical previews were compared against a baseline, specifically the unrelated condition. For the factors Group and Direction, we employed a sum contrast coding method. Participants and items were specified as crossed random effects with random intercepts (Barr et al. 2013). We included the order of target presentation as a covariate since it occurred twice during the experiment. When we ran the models, we always began with full models that included the maximum random effects structure. However, the slopes were removed if the model failed to converge (indicating over‐parametrization). The final model that converged included random intercepts for both subjects and items, along with a random slope across subjects. The *p*‐values were estimated using the “lmerTest” package with the default Satterthwaite's method for degrees of freedom and *t*‐statistics (Kuznetsova et al. 2017). The final converged model is given below:

$$\begin{array}{c} \text{Durations}\sim \text{orderTarget}+{\text{direction}}^{*}\;{\text{group}}^{*}\;\text{preview}\\ +\left(1+\text{direction}\;|\;\text{subject}\right)+\left(1\;|\;\text{item}\right) \end{array}$$

#### 2.10.2 FRP Data Analysis

We analyzed FRPs time‐locked to the first fixation onsets on the target words by using LMMs.^4^ The analysis was preregistered (https://osf.io/34u92/), including time windows and selected electrodes. As previous studies mainly selected a time window of 200–280 ms (e.g., Dimigen et al. 2012) or a time window of 180–280 ms (Buonocore et al. 2020), and as Kornrumpf et al. (2016) suggested that the preview positivity emerged earlier than 200 ms after fixation onset, we selected 180–280 ms as the time window of the preview positivity. Furthermore, as many studies observed the early part of the N1 component, we selected 120–160 ms as the time window of the early N1.^5^ As the early N1 effects and preview positivity have a scalp distribution over occipito‐temporal regions, we selected this area as the region of interest (ROI; left occipital‐temporal area, LOT: PO9/PO7, and right occipital‐temporal area, ROT: PO8/PO10) and also included a factor of *Hemisphere* (left vs. right). The same LMM statistics as for eye movements were applied to FRP epochs, except that the factor *Hemisphere* was also included as an additional predictor, coded as a sum contrast. Post hoc analyses were performed to obtain contrasts, and the tests were adjusted using the multivariate *t*‐distribution (mvt) in the *emmeans* package (Lenth 2018; version 1.7.3). Similar to the eye movement measures, we also included the order of target presentation as a covariate. The final models that converged for early N1 and late N1 were different. For the early N1, the random effects incorporated random intercepts for each subject, along with the random slope of the hemisphere across subjects. Additionally, it encompassed random intercepts for each item and the random slope of direction across items. For the late N1, the random effects consisted of random intercepts for each subject and the random slope of preview across subjects. It also incorporated random intercepts for each item and the random slope of direction across items. The following R codes represent the final converged models.

$$\begin{array}{c} \text{Early N1}\sim \text{orderTarget}+{\text{direction}}^{*}\;{\text{group}}^{*}\;{\text{preview}}^{*}\;\text{hemisphere}\\ +\left(1+\text{hemisphere}\;|\;\text{subject}\right)+\left(1+\text{direction}\;|\;\text{item}\right) \end{array}$$

$$\begin{array}{c} \text{Late N1}\sim \text{orderTarget}+{\text{direction}}^{*}\;{\text{group}}^{*}\;{\text{preview}}^{*}\;\text{hemisphere}\\ +\left(1+\text{preview}\;|\;\text{subject}\right)+\left(1+\text{direction}\;|\;\text{item}\right) \end{array}$$

## 3 Results

### 3.1 Display Change Awareness

In the post‐experimental debriefing about the awareness of saccade‐contingent preview manipulation, all participants, except one in the Taiwanese group, were aware of changes of words from the preview to the target. Eight Mainlanders were able to correctly report the positions of previews located in the vertical reading direction, and nine could do so for the horizontal direction. Four Taiwanese correctly reported the position of previews in the vertical direction, and six Taiwanese correctly reported it for the horizontal direction. In addition, the question about the estimated number of previews showed that Mainlanders reported more changes in the horizontal than the vertical direction (*M* = 37.1 vs. 34.7); in contrast, Taiwanese noticed more changes in the vertical than in the horizontal direction (*M* = 26.2 vs. 24.7). These findings indicate that although participants were not able to recognize the previews, the prevalence of display change awareness was similar across the two participant groups, even though almost all of them noticed the saccade‐contingent preview manipulation.

### 3.2 Animal Task Performance

On average, participants detected 97.7% of the animal names contained in the lists (*d'* = 3.92), as shown in Table 5. The *d‐primes* were calculated for each participant and each direction separately. Repeated measures ANOVA on the within‐subject factor *Direction* and the between‐subject factor *Group* showed that *d‐primes* did not differ as a function of either factor (*Direction*, *F*_(1,58)_ = 1.67, *p* = 0.20; *Group*, *F*_(1,58)_ = 1.52, *p* = 0.22; *Direction × Group*, *F*_(1,58)_ = 0.08, *p* = 0.78). These animal task results suggest that readers read the word lists attentively for comprehension in both groups, regardless of reading direction.

**TABLE 5:** Means (and standard deviations, in parentheses) of sensitivity (*d*′) for responses to targets in vertical and horizontal reading directions between Mainlanders and Taiwanese group.

	Mainlander	Taiwanese
Horizontal (SD)	3.78 (0.54)	3.93 (0.50)
Vertical (SD)	3.93 (0.46)	4.02 (0.53)

### 3.3 Eye Movements

**FIGURE 2:**
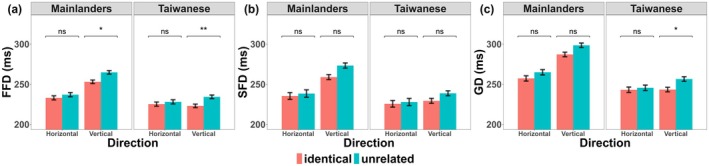
Preview effect on the target word (fixation times following an identical versus unrelated preview). Fixation durations (±1 standard error) are presented separately for FFD, SFD, and GD.

As shown in Figure 2, the eye movement data showed that reading time on the target words following identical previews was shorter than after unrelated previews, confirming the classic preview effect (Table 6). This preview effect was significant for FFD (difference of 8 ms), and GD (difference of 9 ms) but not for SFD (difference of 7 ms). The vertical reading lists required longer fixation durations than the horizontal lists in terms of FFD (difference of 13 ms), SFD (difference of 19 ms), and GD (difference of 19 ms). Taiwanese were generally slightly faster readers than Mainlanders, with shorter FFDs (difference of 20 ms), SFDs (difference of 21 ms) and GDs (difference of 29 ms). However, this was qualified by a significant interaction with Direction for FFD, SFD, and GD, with a larger fixation duration difference between the horizontal and vertical reading directions in Mainlanders than in Taiwanese, as the fixation durations were longer for Mainlanders in the vertical direction than for Taiwanese. In addition, the interaction between Preview and Direction was significant for FFD and a trend for GD (see Table 7 for the results of the linear mixed‐effects model), such that preview effects were larger in the vertical than in the horizontal direction (the preview effects were significant in the vertical direction but not in the horizontal direction). However, the three‐way interaction between Preview, Direction, and Group was not significant for any of the three eye movement measures. The main effect of the order of the target was significant. Therefore, the behavioral data provided no evidence for group differences in preview effects in the two reading directions, although both groups tended to show larger preview effects in the vertical than in the horizontal direction.

**TABLE 6:** Means and standard errors of the fixation time measures (in milliseconds) in the different conditions.

	Condition	FFD	SFD	Gaze
Mainlanders	H‐identical	233 (18)	235 (18)	257 (23)
	H‐unrelated	237 (19)	238 (18)	265 (24)
	V‐identical	253 (14)	258 (13)	287 (20)
	V‐unrelated	265 (16)	273 (15)	299 (21)
Taiwanese	H‐identical	225 (17)	225 (17)	243 (24)
	H‐unrelated	228 (18)	228 (18)	245 (22)
	V‐identical	223 (15)	229 (15)	243 (21)
	V‐unrelated	234 (16)	239 (16)	256 (20)

Abbreviations: FFD, first fixation duration; GD, gaze duration; H, horizontal reading direction; SFD, single fixation duration; V, vertical reading direction.

**TABLE 7:** Fixed effect estimates from the linear mixed‐effects models on the eye movement data.

Factor	First fixation duration			Single fixation duration			Gaze duration		
	*b*	CI	Sign.	*b*	CI	Sign.	*b*	CI	Sign.
(Intercept)	5.41	5.37–5.45	< 0.001***	5.43	5.38–5.48	< 0.001***	5.49	5.45–5.54	< 0.001***
Order	−0.02	−0.03 to −0.00	0.011*	−0.01	−0.03 to 0.01	0.188	−0.02	−0.04 to −0.01	0.006**
Direction	−0.03	−0.05 to −0.01	0.006**	−0.04	−0.06 to −0.01	0.006**	−0.04	−0.07 to −0.02	0.001***
Group	0.04	0.03–0.05	< 0.001***	0.04	0.03–0.06	< 0.001***	0.06	0.05–0.07	< 0.001***
Preview	0.02	0.01–0.04	0.001**	0.02	−0.00 to 0.04	0.056^+^	0.03	0.01–0.04	0.003**
Group × Direction	−0.03	−0.03 to −0.02	< 0.001***	−0.02	−0.04 to −0.01	0.001***	−0.03	−0.04 to −0.02	< 0.001***
Direction × Preview	−0.01	−0.03 to −0.00	0.037*	−0.02	−0.04 to 0.00	0.120	−0.01	−0.03 to 0.00	0.099^+^
Group × Preview	−0.00	−0.01 to 0.01	0.901	0.00	−0.02 to 0.02	0.939	−0.00	−0.01 to 0.01	0.987
Preview × Group × Direction	0.00	−0.01 to 0.02	0.673	−0.00	−0.03 to 0.02	0.626	0.01	−0.01 to 0.02	0.382

^+^ *p* < 0.1.

* *p* < 0.05.

** *p* < 0.01.

*** *p* < 0.001.

### 3.4 EEG Results

**FIGURE 3:**
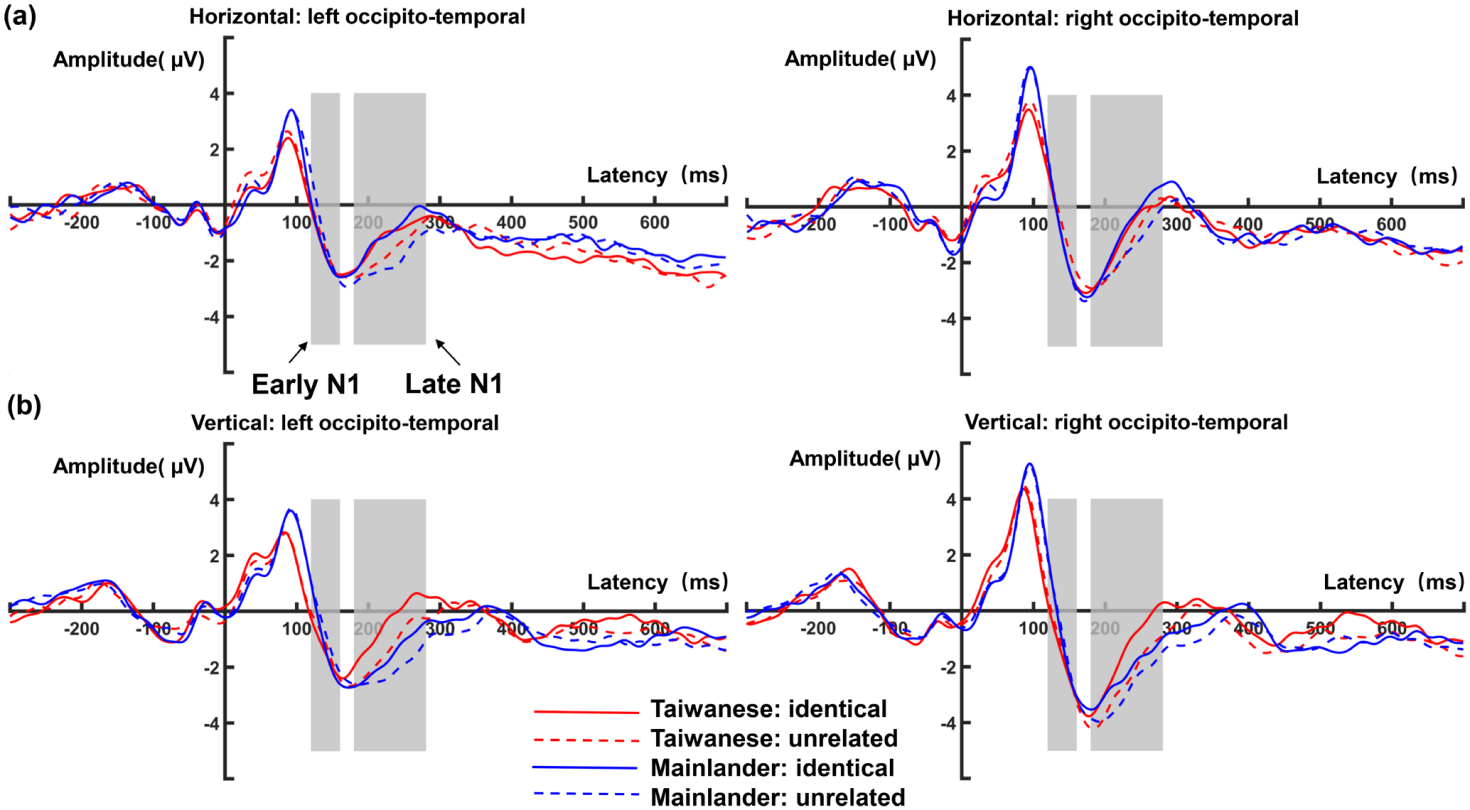
Fixation‐related potential (FRP) waveforms for the horizontal (a) and vertical reading direction (b) at the left and right hemisphere occipito‐temporal regions of interest (LOT and ROT). Gray regions mark the a priori‐defined time windows used for the analyses of early N1 (120–160 ms) and late N1 (180–280 ms).

Figure 3 shows the grand‐average FRPs, time‐locked to the first fixation on target words. The visual inspection of the waveforms at occipital‐temporal electrodes showed the biphasic muscle spike potential around time zero (Keren et al. 2010), followed by a P1‐N1 complex. This complex consisted of the P1 component peaking around 100 ms after fixation onset, and an early part of the N1 component peaking around 170 ms. The early N1 showed larger amplitudes for identical previews than for unrelated ones. After the early N1 peak, the FRP amplitude during the falling flank of the N1 (the late N1) was substantially larger for unrelated previews than for identical ones.

#### 3.4.1 LMM Results (Preregistered Analyses)

##### 3.4.1.1 Early N1 (120–160 ms)

**FIGURE 4:**
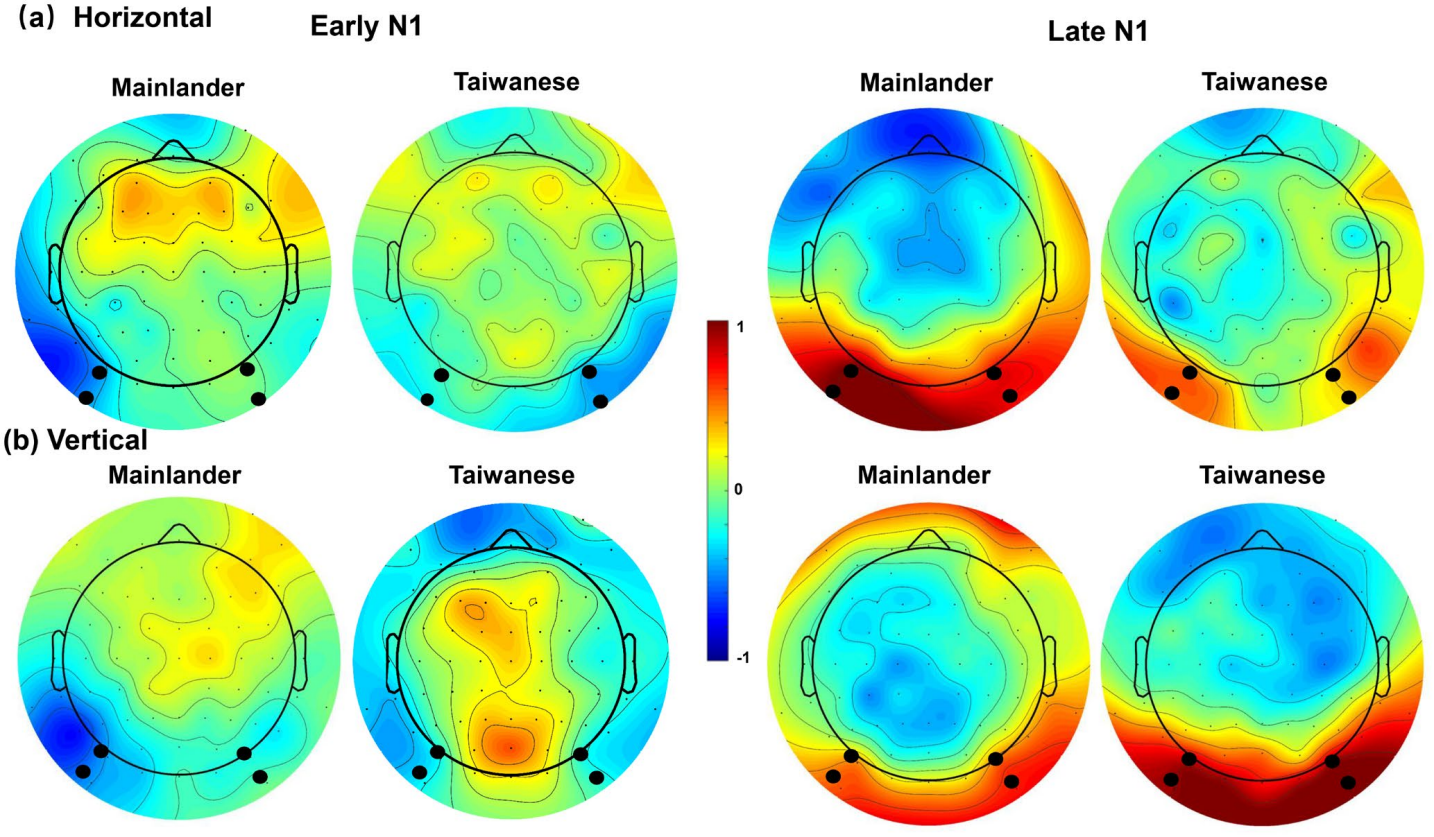
The topographies of the preview effect (identical minus unrelated) for the horizontal (a) and vertical (b) reading direction for Mainlanders and Taiwanese. Topographies are shown for the early N1 (left side) and late N1 (right side). Black dots highlight the electrodes used to define the regions of interest (LOT and ROT).

The early N1 (120–160 ms) tended to be larger (more negative) in the Taiwanese than in the Mainlander group (*b* = 0.18, CI [0.05–0.30], *p* = 0.005). However, the preview effect in the N1 was not significant as a main effect (*Preview*, *b* = 0.25, CI [−0.07–0.57], *p* = 0.12, see Figure 4) nor did it differ between groups (*Preview × Group*, *b* = 0.01, CI [−0.16–0.19], *p* = 0.88) or between the reading directions (*Preview* × *Direction*, *b* = −0.01, CI [−0.26–0.25], *p* = 0.96). The three‐way interaction among *Preview*, *Group*, and *Direction* was also not significant (*b* = 0.01, CI [−0.16–0.19], *p* = 0.89). In addition, the early N1 amplitudes tended to be more negative in the left than in the right hemisphere (*Hemisphere*, *b* = −0.19, CI [−0.39–0.01], *p* = 0.068). However, this was qualified by a significant interaction with reading Direction (*b* = −0.15, CI [−0.27 to −0.03], *p* = 0.014). Post hoc analysis revealed that the scalp distribution was left lateralized in the horizontal direction (*b* = −0.57, CI [−0.99–0.07], *p* = 0.006), but it was bilateral in the vertical direction (*b* = −0.003, CI [−0.38–0.39], *p* = 0.98). All other main effects and interactions were not significant (see Table 8 for the results of the linear mixed‐effects model).

**TABLE 8:** Fixed effect estimates from the linear mixed‐effects models on the EEG data.

Factor	Early N1			Late N1		
	*b*	CI	Sign.	*b*	CI	Sign.
(Intercept)	−1.15	−1.63 to −0.67	< 0.001 ***	−1.31	−1.80 to −0.81	< 0.001 ***
Order	−0.11	−0.39 to 0.16	0.420	0.10	−0.20 to 0.41	0.502
Direction	0.02	−0.16 to 0.20	0.811	0.23	0.07–0.40	0.006**
Group	0.18	0.05–0.30	0.005**	−0.24	−0.35 to −0.12	< 0.001***
Preview	0.25	−0.07 to 0.57	0.124	−0.71	−1.10 to −0.32	< 0.001**
Hemisphere	−0.19	−0.39 to 0.01	0.068^+^	0.21	0.04–0.37	0.015*
Group × Direction	−0.15	−0.27 to −0.02	0.020*	0.28	0.17–0.40	< 0.001***
Direction × Preview	−0.01	−0.26 to 0.25	0.957	0.05	−0.18 to 0.29	0.652
Group × Preview	0.01	−0.16 to 0.19	0.880	−0.01	−0.17 to 0.16	0.936
Direction × Hemisphere	−0.15	−0.27 to −0.03	0.014*	−0.26	−0.37 to −0.15	< 0.001***
Group × Hemisphere	−0.08	−0.20 to 0.04	0.198	−0.08	−0.19 to 0.04	0.196
Preview × Hemisphere	0.08	−0.09 to 0.26	0.336	−0.03	−0.19 to 0.14	0.751
Group × Direction× Preview	0.01	−0.16 to 0.19	0.885	−0.21	−0.37 to −0.05	0.011*
Group × Direction × Hemisphere	0.02	−0.10 to 0.14	0.730	0.04	−0.07 to 0.16	0.447
Direction × Preview× Hemisphere	0.01	−0.16 to 0.19	0.871	−0.06	−0.22 to 0.10	0.491
Group × Preview× Hemisphere	0.11	−0.06 to 0.28	0.201	−0.03	−0.19 to 0.13	0.725
Group × Preview× Direction × Hemisphere	0.05	−0.12 to 0.23	0.534	−0.03	−0.19 to 0.13	0.709

^+^ *p* < 0.1.

* *p* < 0.05.

** *p* < 0.01.

*** *p* < 0.001.

##### 3.4.1.2 Late N1 (180–280 ms)

Our LMM model revealed three significant main effects: Direction (*b* = 0.23, CI [0.07–0.40], *p* = 0.006), Group (*b* = −0.24, CI [−0.35 to −0.12], *p* < 0.001), and Preview (*b* = −0.71, CI [−1.10 to −0.32], *p* < 0.001). We also found a significant interaction between Group and Direction (*b* = 0.28, CI [0.17–0.40], *p* < 0.001), with more negative amplitudes in the Mainlanders group compared to Taiwanese in the vertical direction (*b* = −0.84, CI [−1.05 to −0.63], *p* < 0.001), but not in the horizontal direction (*b* = −0.12, CI [−0.34–0.13], *p* = 0.34). These effects were further explained by a significant three‐way interaction between Preview, Group, and Direction (*b* = −0.21, CI [−0.37 to −0.05], *p* = 0.011). Follow‐up analyses of this three‐way interaction showed distinct patterns between groups: Taiwanese readers demonstrated a significant preview effect only during vertical reading (*b* = 0.96, CI [0.38–1.55], *p* < 0.001), whereas Mainland readers showed this effect only during horizontal reading (*b* = 0.87, CI [−0.10–1.64], *p* = 0.018). Additionally, we observed a significant main effect of Hemisphere (*b* = 0.21, CI [0.04–0.37], *p* = 0.015), which interacted significantly with reading direction (*b* = −0.26, CI [−0.37 to −0.15], *p* < 0.001). This interaction revealed that neural activity was bilateral during horizontal reading (*b* = −0.19, CI [−0.53–0.15], *p* = 0.27) but right‐lateralized during vertical reading (*b* = 0.96, CI [0.64–1.28], *p* < 0.001). No other effects, including Order, reached statistical significance.

#### 3.4.2 Exploratory Analysis

To further investigate whether there were effects in the FRPs beyond the time windows and electrodes that we had selected a priori, we used a sample‐by‐sample Topographic Analysis of Variance (TANOVA) that included all electrodes in the FRP map. The Ragu software (Koenig et al. 2011) was used on non‐normalized (raw) topographic maps to test for effects of the two within‐subject factors (*Direction* and *Preview*) and the between‐subject factor (*Group*). The TANOVA was corrected for multiple comparisons through Global Duration Statistics (Koenig et al. 2011). If this global test was significant (*p* < 0.05 at 5000 randomization runs), *t*‐maps (across participants and against zero) of the covariance maps were computed and displayed. As we were mainly interested in preview effects corresponding to the early N1 and late N1 effects, we focused on the first 400 ms after fixation onset.

**FIGURE 5:**
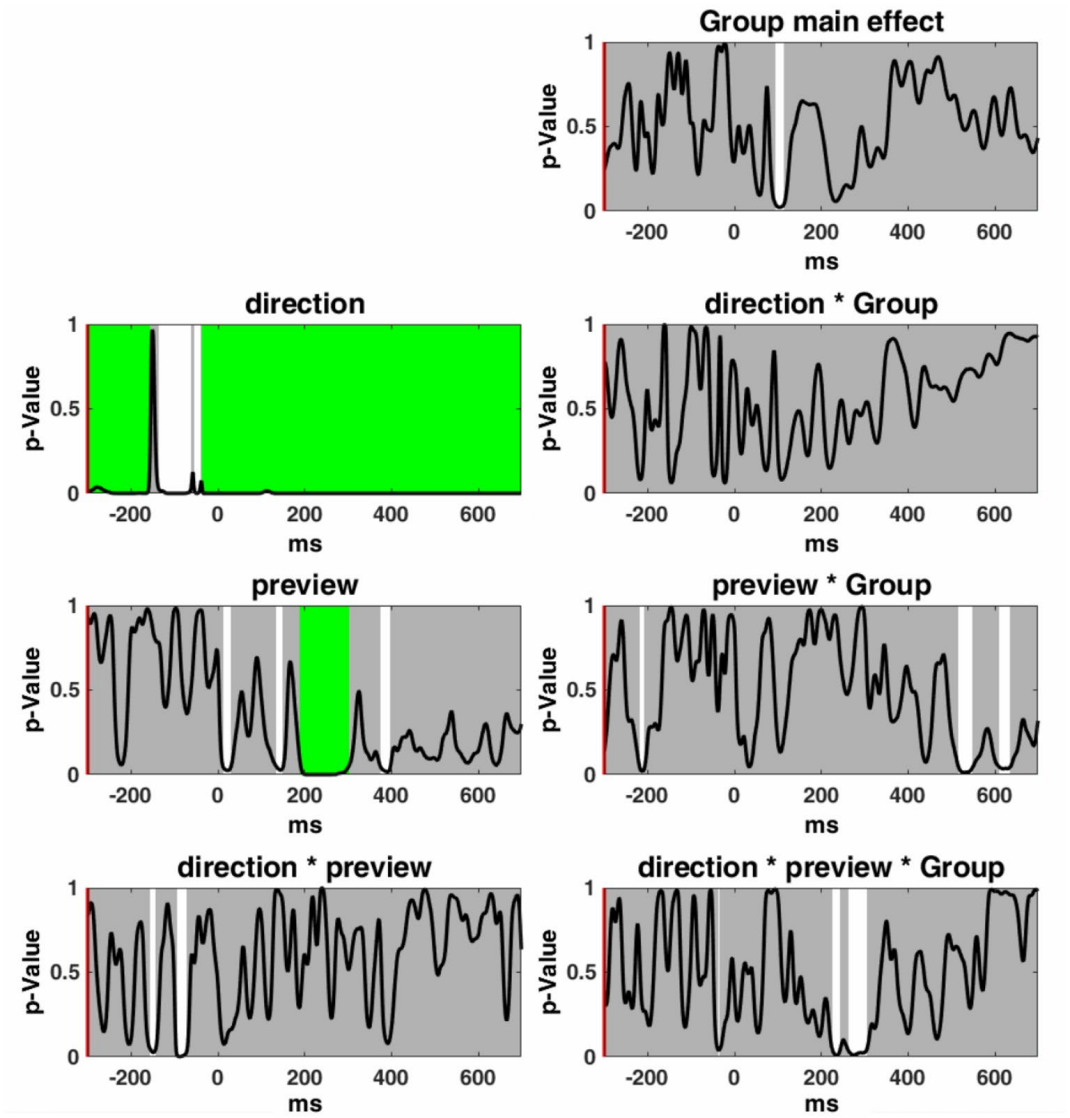
Results of the exploratory sample‐by‐sample TANOVA for the different factors and contrasts. Each plot visualizes the *p*‐values (*y*‐axis) for the comparison between the mean FRP maps of each factor level or interaction at every time point after fixation onset (milliseconds on the *x*‐axis). Gray areas mark nonsignificant time points, whereas the white areas mark periods of significant differences between the factor levels. We corrected for multiple comparisons using global duration statistics, and the duration thresholds were then applied to the TANOVA plots. These periods longer than the estimated duration threshold are marked in green (i.e., effects corrected for multiple comparisons). For the main effect of *Direction*, the duration threshold was identified to be 82 ms. For the main effect of *Preview*, the duration threshold was identified to be 45 ms.

As shown in Figure 5, the main effect of *Direction* was significant across the entire time window after fixation onset,^6^ and the preview effect was significant in the time window of 191–303 ms. The main effect of *Group* was significant between 93 and 115 ms, which may correspond to the P1 component. Given the temporal proximity and potential overlap between the P1 and the early part of N1 components, the group differences we observed in the early N1 (in the LMM model) may actually reflect the residual effects originating from the preceding P1 component or a faster transition between the P1 and N1 components. This temporal relationship suggests caution in interpreting the early N1 effects independently from P1 activity. The three‐way interaction among *Preview*, *Direction*, and *Group* reached significance during the time window of 225–305 ms (separated by a short interval of 15 samples where *p*‐values were smaller than 0.1), although the two time windows did not survive correction for multiple comparisons. The other interactions were not significant within 400 ms after fixation onset. Overall, the TANOVA results were consistent with the ROI analysis of the late N1, especially as the time windows identified by TANOVA for the preview effect and the three‐way interaction, had a large overlap with the ROI analysis. However, for the early N1 component, the time window identified for preview effects (135–147 ms) did not survive the multiple comparison correction, and no significant time window was identified for the three‐way interaction (*Preview × Direction × Group*). In addition, two small time windows were identified for the two‐way interaction of Preview and Group around 600 ms and one small time window was identified around 400 ms for the preview effect; however, they were not sustained after multiple comparison correction. The results were expected, as our study manipulated the visual forms of the stimuli and used word lists, semantic activation, and other higher‐level processes, which were less robust compared to using sentences as materials (e.g., Li et al. 2015, 2024).

## 4 Discussion

For the first time, the present study investigated the influence of experience in reading in different directions, namely, horizontal and vertical, on neural correlates of visual word recognition. We recruited participants from Taiwan and mainland China, who spoke Mandarin, and tested them on the same reading materials, presented in both horizontal and vertical directions. We used a boundary paradigm together with the co‐registration of EEG and eye movements, allowing readers to move their eyes freely as in natural reading. In the boundary paradigm, either identical or unrelated previews were presented. We expected that the preview effects would differ as a function of different reading directions between the two groups of readers, especially in the vertical direction, due to the much more pervasive experience of Taiwan residents with vertical script.

Results replicated several common observations in behavior. First, we found preview effects in eye movements. Furthermore, both groups performed very accurately in the animal detection task, suggesting that reading performance was good and not significantly different between groups. More importantly, not only did we find the typical preview positivity, but our results also demonstrate for the first time that the presence of a preview positivity in the vertical reading direction depends on readers' prior experience with reading in that direction. The main findings of the preview effects in both eye movements and FRP, and how vertical reading experiences modulate preview effects, are discussed below.

### 4.1 Preview Effects in FRP

The analysis of FRPs showed a reduced occipito‐temporal negativity after identical preview in the time window from 180–280 ms, which we called late N1 (see also Kornrumpf et al. 2016), which in previous papers has also been called “preview positivity” (Dimigen et al. 2012) because of the more positive‐going amplitudes after valid previews. Additionally, we also obtained this preview effect in TANOVAs, where the significant time windows overlapped with the ones we had preregistered, suggesting that the effect is robust and can be detected across the entire map. The preview positivity is usually considered to reflect the preview‐based facilitation of early stages of visual word recognition at orthographic and/or phonological levels (Niefind and Dimigen 2016). The effect's time course and scalp distribution (largest over left occipito‐temporal regions) fits to previous late N1 or N250 findings (e.g., Bentin et al. 1999; Maurer et al. 2005), which have been linked to orthographic processing. In a previous study (Huang et al. 2025), we showed that the late N1 in masked priming and boundary paradigms may reflect similar neural mechanism, which is sensitive to the visual‐orthographic properties of words.

However, contrary to our hypothesis, we did not obtain an electrophysiological preview effect for the early part of the N1 component (preregistered as the interval between 120–160 ms), although there was a trend showing that identical words were more negative than unrelated words in FRP amplitudes. Although the early N1 preview effect was rarely reported in the FRP literature (e.g., Degno et al. 2019a; Dimigen et al. 2012), an N1 effect with larger negativity for primed vs. unprimed words is frequently seen in masked priming studies (Chauncey et al. 2008; Huang et al. 2022). Therefore, this early N1‐like preview effect, similar to the N1 effects in the masked priming paradigm, may reflect the initial stage of sublexical orthographic processing in visual word recognition (Grainger and Holcomb 2010), which is sensitive to the degree of visual overlap between previews and targets. However, as the preview effect in the early N1 component was only marginally significant, the initial stage of sublexical orthographic processing may not be as robustly modulated by parafoveal previews as the later stage (reflected in the late N1) of visual word recognition.

Additionally, TANOVA revealed N400 preview effects, though these did not survive multiple comparison correction. As detailed in the Supporting Information, the structural properties of Chinese characters position semantic radicals closer to the fovea during both horizontal and vertical reading, facilitating sub‐lexical semantic processing (e.g., Yan, Risse, et al. 2012). However, semantic processing may have been attenuated in our study because of the absence of sentential context.

### 4.2 Does Experience in Reading Direction Modulate Preview Effects in FRP?

The key finding of our study is the three‐way interaction among Preview, Group, and Direction for the late N1, which demonstrates how reading experience shapes parafoveal processing. Our questionnaire confirmed that Taiwanese participants had extensive experience with vertical reading, whereas Mainland participants predominantly read horizontally. This differential reading experience was reflected in distinct preview effects: in the vertical direction, Taiwanese readers showed larger preview effects compared to Mainland readers, whereas in the horizontal direction, Mainland readers demonstrated larger preview effects compared to Taiwanese readers. These findings differed from our initial hypothesis, where we expected similar preview effects across both groups in the horizontal direction because of their shared experience with horizontal text. However, our questionnaire data revealed clear between‐group differences in reading experience—Taiwanese readers reported more extensive vertical reading experience compared to Mainland readers, whereas both groups had substantial horizontal reading experience. This expertise pattern was mirrored in our experimental results, with each group showing processing advantages in the reading direction they had more experience in, compared to the other group. The robustness of this finding was confirmed by both ROI analysis and TANOVAs, indicating a widespread effect across the scalp. These results suggest that readers process parafoveal information more efficiently in the reading direction they have more expertise in, compared to the other group: Taiwanese readers excelled in vertical reading compared to Mainland readers, whereas Mainland readers showed advantages in horizontal reading compared to Taiwanese readers.

The timing of the preview positivity (in the late N1) aligns with previous studies demonstrating expertise effects in the N170 component across various domains, including bird experts viewing birds (Tanaka and Curran 2001), car experts viewing cars (Gauthier et al. 2003), fluent readers processing printed words (Maurer et al. 2005), and non‐prosopagnosic observers viewing faces (Rossion and Jacques 2012). Our findings extend this research by showing how extensive experience with a particular reading direction enhances processing efficiency, specifically for the direction that received more practice relative to the other group. More specifically, the two groups could have different levels of word form familiarity on the basis of their respective reading orientations. The visual appearance of two‐character words differs fundamentally between vertical and horizontal orientations due to their distinct spatial arrangements. Taiwanese readers, with extensive exposure to vertical text, showed processing advantages for vertically aligned words, whereas Mainland readers, with predominant exposure to horizontal text, demonstrated better processing for horizontally aligned words. These processing advantages likely reflect each group's familiarity with word forms in their more practiced direction relative to the other group, contributing to the observed three‐way interaction.

An alternative explanation for the three‐way interaction could be that the two groups have different levels of word form familiarity on the basis of their distinct reading experiences. Since the two‐character words were arranged differently in the vertical and horizontal directions, their visual word forms varied across orientations. Taiwanese readers, who have substantial exposure to both text arrangements but particularly vertical ones, showed processing advantages for vertically aligned words, whereas Mainland readers, with predominant exposure to horizontal text, demonstrated better processing for horizontally aligned words. These processing advantages likely reflected each group's familiarity with word forms in their frequently encountered reading directions. This differential familiarity with visual configurations may have contributed to the observed three‐way interaction, where each group showed enhanced preview effects in the reading direction they had extensive experience with.

Contrary to our expectations, we found no significant interactions between the early N1 preview effects with Group or Direction, possibly because of the nonsignificant early N1 preview effect itself. This suggests that at the earliest stages of visual word recognition, reading direction experience may not significantly influence processing. The results indicate that during initial visual processing, neither the stimulus orientation nor readers' directional reading experience substantially affects word recognition.

### 4.3 Lateralization for Reading Direction?

We also obtained an interaction between *Direction* and *Hemisphere*, both in the early and late N1, such that the vertical direction showed a right‐hemisphere bias, but the horizontal direction showed a slight tendency toward left‐lateralization in both groups. Previous literature has found that the reading‐related N170 is left‐lateralized (Bentin et al. 1999; Maurer et al. 2005; Tarkiainen et al. 1999), with larger amplitudes over the left hemisphere for words than for low‐level visual stimuli. This left‐lateralized N170 topography elicited by visual words stands in contrast to N170 responses for other forms of perceptual expertise related to faces or objects of expertise, which are typically bilateral or right‐lateralized (Rossion et al. 2003; Tanaka and Curran 2001). Previous hypotheses suggested that left‐lateralization of the N170 was due to the involvement of phonological processing during learning to read (phonological mapping hypothesis; Maurer and McCandliss 2007) or due to a larger degree of high spatial frequencies in visual words (spatial frequency hypothesis, Mercure et al. 2008). However, neither of these two hypotheses can explain the current findings with left‐lateralization only for the horizontal direction, as phonological influences and spatial frequencies were the same for the two reading directions. This finding is potentially very interesting and suggests another or additional mechanism that may explain left‐lateralized processing of visual words.

The left‐lateralization for visual words is considered to be part of the left hemisphere language network, and this left‐hemispheric dominance for language has been found to be not only associated with alphabetic languages (e.g., Brem et al. 2006; Cohen et al. 2000), but also logographic languages (i.e., Japanese, Maurer et al. 2008; Chinese, Tan et al. 2001; Xue et al. 2019). However, evidence for left‐lateralization for printed script is usually derived from writing systems in which the text runs from left to right, whereas for scripts with a right‐to‐left orientation, the lateralization is sometimes right‐biased, but further neuroimaging evidence is lacking (e.g., Hebrew, Yiddish, for a review, see Obler 1989; but see also Orbach 1952). Therefore, the right‐laterization for words during vertical reading in the current study may suggest that left hemisphere activation for visual‐orthographic information may be related to left‐to‐right reading, and therefore could be related to eye movements and attention allocation. Since in left–right reading, the parafoveal information is located in the right visual field, this may further influence visuospatial attention and oculomotor behavior. However, further investigation is needed to test these hypotheses.

### 4.4 No Modulation of Behavioral Preview Effects by Vertical Reading Expertise

Consistent with the FRP analysis, the eye movement data also showed typical preview effects in the vertical direction, as the preview effects were significant in both FFD and GD, consistent with previous reports (Degno et al. 2019a, 2019b; Dimigen et al. 2012; Kornrumpf et al. 2016). Preview effects in fixation times were small (e.g., 8 ms in FFD) compared to those in previous studies (e.g., FFD: 20 ms in Dimigen et al. 2012; 26 ms in Kornrumpf et al. 2016; 38 ms in Dimigen and Ehinger 2021; 41 ms in Degno et al. 2019a; 35 ms in Yan et al. 2009; 14 ms in Yang et al. 2009; see Tsang and Chen 2012 for a review). The small size of the preview effect in the current study may be at least partly due to the use of word lists rather than sentences as materials, especially as we used Chinese word materials. In typical Chinese text reading, words are presented without spaces in between. However, in the present study, we inserted spaces between words, and previews were positioned at a 5.5° visual angle. In contrast, the majority of studies exploring preview effects in Chinese that used sentences (e.g., Yan, Risse, et al. 2012; Yang et al. 2009; Yen et al. 2008), positioned the invisible boundary between unspaced words, resulting in previews with visual angles of 2–3 degrees. Given that visual acuity decreases as the visual angle increases, the relatively large visual angle in the present study might account for the relatively small preview effects in the EM data compared to other studies. Since we employed word lists as materials, predicting upcoming words on the basis of sentence context was not feasible, which might have enhanced preview effects during sentence reading.

In addition, the changes in the visual font employed here may have influenced the size of the preview effect. We used different fonts for previews and targets to reduce visual overlaps. Previous priming studies have found that changes in font between primes and targets modulated early visual components (i.e., the early and late N1 components; Chauncey et al. 2008; Zhou et al. 2016). Therefore, changes between previews and targets may have introduced additional variance, diminishing the preview effects. We also found that both Taiwanese and Mainlanders fixated longer during vertical reading than horizontal reading. This finding is consistent with previous reports, as Yan et al. (2019) found that Taiwanese readers showed longer fixation durations in vertical than horizontal reading. A similar finding was obtained for readers without expertise in vertical reading (Laarni et al. 2004), indicating that vertical reading experiences may not modulate fixation durations on left–right and top‐down reading directions. The generally longer fixation durations in the vertical direction, compared to the horizontal direction, might be associated with a smaller perceptual span in the vertical direction (Yan et al. 2024). This is because a smaller perceptual span typically correlates with slower reading speed (Yu et al. 2010). Lower‐level sensory factors, such as crowding, positional uncertainty, and alterations in peripheral acuity, influence the perceptual span (for a review, see Legge 2007, Ch. 3). Also, visual acuity is better in the horizontal than in the vertical meridian (asymmetries in visual acuity around the visual field), which could further contribute to a smaller perceptual span in the vertical direction.

Furthermore, some biological factors may also have an influence on the reading direction effects. For example, the spatial density of photoreceptor cells along the horizontal direction is generally higher than along the vertical direction (Curcio et al. 1990). Similarly, Najemnik and Geisler (2008) found that target visibility drops faster vertically than horizontally. Furthermore, evidence on the neurological control of horizontal and vertical eye movements has shown that vertical saccades are slower than horizontal saccades, and that the downward saccades are the slowest (Terry Bahill and Stark 1975). Therefore, the biological basis and the neural control of the visual system may influence reading in the vertical and horizontal directions of texts.

On the other hand, the generally longer fixation durations in the vertical direction, compared to the horizontal direction, could account for the observed preview effects in the vertical direction in both groups. A study by Yan, Risse, et al. (2012) found that preview effects increase with longer preview durations. Hence, given the increased fixation durations in the vertical direction, the likelihood of observing preview effects may also increase. The eye movement data did not show the critical three‐way interaction among *Preview*, *Direction*, and *Group*, which we observed in FRPs. The absence of the three‐way interaction in eye movements is likely a consequence of the numerically small preview effects in the groups for both directions. Although the three‐way interaction was not significant, the two‐way interaction between *Group* and *Direction* was, as Taiwanese readers had shorter fixation durations in vertical directions than Mainlanders, indicating that vertical reading expertise modulates fixation durations. The discrepant findings regarding the three‐way interactions in EM and FRPs will be discussed further below.

Additionally, we obtained a main effect of *Group*, with Mainlanders showing overall longer fixation durations than Taiwanese, although comprehension performance (as indicated by the performance in the animal task) was not significantly different. This overall group effect cannot be explained by the faster vertical reading in the Taiwanese group, as further tests for each reading direction showed longer fixation durations for Mainlanders than Taiwanese, even in the horizontal direction. A possible reason may be that readers who use the simplified Chinese system (i.e., Mainlanders) processed the text in a less holistic way than traditional Chinese readers (i.e., Taiwanese) when perceiving characters (Liu et al. 2016), Mainlanders may be more sensitive to internal constituent components of characters and may need more time for recognition. Such long‐term influences of reading and writing experience with the two writing systems cannot be ignored, although the materials we selected had the same visual forms in both simplified and traditional Chinese.

### 4.5 On the Relation of Eye Movement and FRP Data

We observed some discrepancies between eye movement and FRP findings, particularly in the three‐way interactions among Preview, Direction, and Group. Specifically, in eye movements, the preview effect was only evident in the vertical direction for both groups, whereas in the late N1 of the FRP, each group showed preview effects only in the direction for which they had dominant reading experience. These findings might suggest that the reading experiences differentially influence neural activity and oculomotor behavior in vertical and horizontal reading. The absence of the late N1 preview effect in vertical reading in Mainlanders, although they show preview benefits, may be because of a later origin of their performance effects than the late N1 processes. This is, however, not supported by ERPs because we did not find post‐N1 preview effects in the TANOVA. Alternatively, the discrepancy between preview effects in eye movement and FRP might be due to differential sensitivity. Thus, the lack of vertical N1 preview effects in Mainlanders might be due to increased neural variability in their non‐expertise reading direction. Similarly, the absence of preview benefits in horizontal reading in Taiwanese may also be due to increased neural variability in their neural data. Regardless of which explanation is correct, the FRP data indicated expertise effects in the preview effect for late N1 stage processes.

The apparent discrepancy between eye movement and EEG findings may reflect their different temporal sensitivities. Although ERP/FRP effects can indicate when certain cognitive processes might begin to influence reading, eye movement measures reflect the combined outcome of multiple processes, including those of oculomotor preparation. In our study, reading expertise modulated the preview effect in the late N1 time window, which has been associated with aspects of visual word processing. Although this expertise‐related modulation was observed in the earlier time window of the EEG effect, it was not detected in the eye movement measures. Our results highlight the complex temporal dynamics between different aspects of visual processing during reading, where effects observed in comparatively earlier EEG time windows may not directly translate to behavioral measures that reflect only the culmination of multiple processing stages.

We believe that the discrepancy we observed between EM and FRP results underscores the value of co‐registration methods in reading research. Although we found no typical preview effects in horizontal reading for either group in the EM data, the FRP results revealed distinct patterns of neural activity. This divergence between behavioral and neural measures demonstrates why relying on either method (EM or ERP) alone could provide an incomplete picture. Our findings align with previous research (Milligan et al. 2023) showing that combining eye‐tracking and EEG can yield complementary insights unavailable through single‐method approaches. The co‐registration technique proved particularly valuable in our study by revealing how eye movement control and cognitive lexical processes may integrate differently in reading, especially given that most EM models emphasize eye movement control over word processing (e.g., the SWIFT model, Engbert et al. 2005; Glenmore, Reilly and Radach 2002). However, given the unexpected lack of preview effects in our EM data, we recommend further replication with methodological refinements that may include an increased sample size, consistent fonts, the removal of word spaces, reduced visual angles, and natural sentence reading scenarios.

### 4.6 Limitations

Our study also has several potential limitations. First, we noted that the number of accepted trials for the FRP analysis differed between the reading directions, with more remaining data for the vertical direction (because of a smaller number of failed fixation checks at the beginning of the trial). However, we believe that the fewer trials in the horizontal direction cannot explain the critical three‐way interaction, as the two groups did not differ from each other with regard to the trial number in a given reading direction. Especially for the FRP analysis, no two‐way interaction of *Group* and *Direction* was found, and Mainlanders showed a preview effect only in the horizontal but not in the vertical direction, suggesting no weakening of the preview effect in the horizontal direction.

Second, the differences in writing systems may have an impact on readers' character perception. Consistent evidence has shown greater visual discrimination skills in readers of simplified rather than traditional Chinese (McBride‐Chang et al. 2005; Peng et al. 2010; Yang and Wang 2018) and more analytic character processing (Liu et al. 2018). Therefore, readers of the two writing systems may process the same characters in different ways, which may further influence the neural correlates of character processing. However, this cannot explain the three‐way interaction, as such an effect should be independent of the reading direction.

Third, all participants in the current study were living in Hong Kong at the time of the study, where the horizontal reading direction is mainly used. Our Taiwanese participants had therefore presumably less exposure to vertical text compared to readers who were residing in Taiwan more recently. The observed preview effects in the vertical direction for the Taiwanese group in the current study may therefore be a conservative estimate of the influence of the experience with a reading direction, and it may also partially explain the absence of the three‐way interaction among *Group*, *Direction*, and *Preview* in the eye‐movement data. Further studies may address this issue by recruiting participants who were not recently exposed to a different proportion of reading directions.

Fourth, as mentioned earlier, we used different fonts for previews and targets. The change of font between preview and target words was designed to serve two purposes: (1) to avoid visual overlap between previews and targets, and (2) to prevent participants from using font type as a cue to predict upcoming target words. However, this manipulation may have introduced an unintended confound in attention distribution. Specifically, in the right/lower visual field, participants consistently encountered font changes, which may have increased cognitive load. This increased load could have affected both oculomotor behavior and the depth of parafoveal preprocessing (Reichle 2011).

Finally, we manipulated previews using valid and invalid conditions, an approach that is common in the literature (e.g., Li et al. 2015; Rayner 1975; Risse and Seelig 2019; Risse and Kliegl 2014; Veldre and Andrews 2018). However, this design makes it difficult to disentangle visual from language processing. A future study that includes a non‐word control condition would be informative for addressing this limitation.

## 5 Conclusions

The present study provides the first evidence that readers with vertical reading expertise show a larger preview positivity in their fixation‐related brain activity compared to readers who are less accustomed to vertical text. This modulation of preview effects by the reader's cultural experience indicates that long‐term reading experience in the vertical direction shapes how readers process written words. The results also shed light on how a person's everyday visual experience influences parafoveal processing. Furthermore, we found that EM and EEG provide distinct but complementary information on how oculomotor and neural activity interact and integrate. Future studies may consider the potential influences of reading experiences on different stages of visual word processing and their reflection in oculomotor and neural processing.

## Author Contributions

Xin Huang: Conceptualization, data curation, formal analysis, investigation, and writing – original draft. Hezul Tin‐Yan Ng: Investigation, data curation, and software. Chien Ho Lin: Conceptualization, investigation, and resources. Ming Yan: Conceptualization and writing – review and editing. Olaf Dimigen: Methodology, writing – review and editing, and software. Werner Sommer: Funding acquisition, conceptualization, and writing – review and editing. Urs Maurer: Funding acquisition, supervision, conceptualization, and writing – review and editing.

## Funding

This work was supported by the Hong Kong Special Administrative Region Research Grants Council, G‐CUHK409/18. German Academic Exchange Service, Project 57447990.

## Conflicts of Interest

The authors declare no conflicts of interest.

## Supporting information


**Supplementary Table 1.** The number of characters in different structures.
**Supplementary Table 2**. Results of ANOVA on the EEG data.

## Data Availability

The data that support the findings of this study are openly available in the Open Science Framework at https://osf.io/34u92/.

## Endnotes

^1^
Please note that the word frequencies for the same words in Sinica Corpus are usually lower than in SUBTLEX‐CH corpus.

^2^
We selected 5.5° as the visual angle of preview, on the basis of timing tests for 5 different visual angles (ranging from 4–6°, in 0.5° increments), to make sure that the display change can be triggered with minimal error.

^3^
Results from a post hoc power analysis conducted using PANGEA (v0.2; Westfall 2015) indicated that with around 60 items per condition and 30 participants per group, the current study design would be capable of detecting a medium effect size (*d* = 0.50; e.g., Milligan et al. 2023) for the main effect of preview at a power level of 0.99.

^4^
We switched the analysis from ANOVA to LMM as we wanted to make the results of EM and FRPs more comparable, so the LMM method was adopted for both EM and FRP measures. The results of ANOVA analysis are provided in the Supporting Information.

^5^
Note that previous papers did not always differentiate between the early N1 and the preview positivity in their analysis, but sometimes referred to the preview positivity as the “late parts” or “falling flank” of the N1 component (Kornrumpf et al. 2016). Our current analysis distinguished between these two intervals. The two components were distinguished by the direction of preview effects.

^6^
We noted that for the main effect of *Direction*, all *p*‐values were smaller than 0.05 after fixation onset, whereas in the ROI analysis, the main effect of Direction was not significant. Possibly, the selection of electrodes in the ROI cannot explain the direction differences as the selection of the ROI was based on preview effects. Further t‐maps on the effect of *Direction* showed that the largest activation in the corresponding time windows of the early and the late N1 was located in the eye electrodes and central‐posterior sites, thus, the occipito‐temporal electrodes may not reflect the *Direction* effect.
