# Supplementary material for: How the Dominant Reading Direction Changes Parafoveal Processing: A Combined EEG/Eye‐Tracking Study
Source: Psychophysiology. 2025 Dec 4;62(12):e70205. doi: 10.1111/psyp.70205 (PMC12678225; doi:10.1111/psyp.70205)
Supplement: Supplementary file 1 — Supplementary Table 1. The number of characters in different structures. Supplementary Table 2. Results of ANOVA on the EEG data. [file PSYP-62-e70205-s001.docx]

## **Supplementary materials**

1. **Results of reading experience questionnaire**

A self-report questionnaire was administered to evaluate participants' reading and writing experiences in horizontal and vertical directions before and after moving to Hong Kong. Participants reported their exposure to vertically and horizontally aligned texts across 10 different media types: magazines, books, comics, newspapers, textbooks, smart device content, road signs, billboards, slogans/leaflets, and advertisements (using a 0-4 scale, where 0=never and 4 = always). Reading time for different text categories was also evaluated. Additionally, participants reported their age of first exposure to horizontal and vertical text directions, as well as their writing experiences in both orientations.

Before moving to Hong Kong, repeated measures ANOVAs revealed a significant main effect of Direction and a significant Direction × Group interaction across different text categories (*F*s > 13.34, *p*s < 0.001), but no significant main effect of Group (*F*s < 1.31, *p*s > 0.25). Post-hoc t-tests showed that Mainlanders reported more experience reading horizontal texts than Taiwanese participants (including textbooks, slogans, leaflets, road signs; *t*s > |2.92|, *p*s < 0.05), whereas Taiwanese reported more experience reading vertical texts compared to Mainlanders (including textbooks, *t*s > |3.48|, *p*s < 0.001). Within-group comparisons revealed that Mainlanders had significantly more horizontal than vertical reading experiences (*t*s > 9.94, *p*s < 0.001), while Taiwanese exhibited similar reading experiences in both directions (*t*s < 1.78, *p*s > 0.29). Additionally, 9 Taiwanese participants reported converting digital texts to vertical direction through apps, whereas 7 Mainlanders reported converting texts from vertical to horizontal direction.

After moving to Hong Kong, repeated measures ANOVAs again revealed a significant main effect of Direction and a significant Direction × Group interaction across different text categories (*F*s > 85.61, *p*s < 0.001), but no significant main effect of Group (*F*s < 2.01, *p*s > 0.16). Post-hoc between-group comparisons showed that Taiwanese and Mainlander participants had similar vertical reading experiences (*t*s < 2.02, *p*s > 0.19), except for general reading, where Taiwanese had more vertical experiences than Mainlanders (*t*(58) = -3.17, *p* = 0.02). Mainlanders maintained more horizontal experience than Taiwanese (including textbooks and road signs, *t*s > |3.15|, *p*s < 0.003, but not for slogans and leaflets, *t*s < |1.65|, *p*s > 0.11). Within-group t-tests showed that both Mainlanders and Taiwanese had more experiences in the horizontal direction compared to vertical direction (Mainlanders: *t*s > 7.16, *p*s < 0.001; Taiwanese: *t*s > 3.96, *p*s < 0.001), except for textbook reading among Taiwanese, where experiences between directions were similar (*t*(58) = 2.00, *p* = 0.20).

The total reading time for both groups was similar before and after moving to Hong Kong across most text categories (books, comics, magazines, and smart device content; *F*s < 1.11, *p*s > 0.29), except for newspapers, where Taiwanese spent more time reading compared to Mainlanders before moving to Hong Kong (*t*(58) = -2.09, *p* = 0.04).

Regarding the age of first exposure to different text directions, significant main effects of Group and Direction were found, along with a significant Group × Direction interaction (*F*s > 27.36, *p*s < 0.001). Post-hoc t-tests showed that Taiwanese were exposed to vertical text at an earlier age than Mainlanders (5.2 vs. 10.85 years old, *t*(58) = 7.50, *p* < 0.001), whereas Mainlanders were exposed to horizontal text earlier than Taiwanese (3.5 vs. 4.5 years old, *t*(58) = 3.73, *p* < 0.001). Within-group comparisons revealed that Mainlanders learned to write horizontally significantly earlier than vertically (*t*(58) = -12.46, *p* < 0.001), whereas Taiwanese learned to write in both directions at a similar age (*t* = -0.045, *p* = 0.97).

For writing experiences in each direction, significant main effects of Group and Direction were found, along with a significant interaction (*F*s > 9.77, *p*s < 0.003). Between-group comparisons showed that Taiwanese participants reported more vertical writing experience than Mainlanders before moving to Hong Kong (*t*(58) = 5.00, *p* = 0.001), but not after (*t*(58) = -2.02, *p* = 0.19). Conversely, Mainlanders reported significantly more horizontal writing experience than Taiwanese both before and after moving to Hong Kong (*t*s > |3.57|, *p*s < 0.004). Within-group comparisons showed that Mainlanders reported more horizontal than vertical writing experiences both before and after moving to Hong Kong (*t*s > 15.31, *p*s < 0.001), whereas Taiwanese reported more vertical than horizontal writing experiences both before and after moving (*t*s > 9.66, *p*s < 0.001).

1. **Structures of Chinese characters**

Chinese characters have ten main structural types: single-radical, left-right, up-down, up-left, up-right, left-down, up-left-down, left-up-right, left-down-right, and enclosure (Dai et al., 2007). As requested by the reviewer, we analyzed the structures of the first character in our preview/target words to determine whether certain structures position radicals closer to the fovea. Since the preview words were also served as targets in another word lists, each character was only calculated once. As shown in the table (supplementary Tabel 1), most characters (77.6%) were either left-right or up-down structures. We discussed how the structures may influence preview effects in different directions.

Supplementary Table 1. *The number of Characters in different structures*

| Structure | Number of Characters |
| --- | --- |
| Single-radical | 17 |
| Left-right | 71 |
| Up-down | 40 |
| Up-left | 8 |
| Up-right | 2 |
| Up-left-down | 1 |
| Left-down-right | 4 |
| Enclosure | 1 |

1. **Supplementary Results for ANOVA results on the EEG data**

Supplementary Table 2. *Results of ANOVA on the EEG Data*

| Factor | Early N1 | | | |  | Late N1 | | |
| --- | --- | --- | --- | --- | --- | --- | --- | --- |
|  | *F* | Sign. | Partial  eta square |  | *F* | Sign. | Partial  eta square |  |
| Direction | 0 | 0.98 | 0 |  | 4.31 | 0.042* | 0.07 |  |
| Direction × Group | 1.48 | 0.23 | 0.03 |  | 2.46 | 0.12 | 0.04 |  |
| Preview | 6.38 | 0.014* | 0.10 |  | 40.54 | <0.001*** | 0.41 |  |
| Preview × Group | 0 | 0.99 | 0 |  | 0.03 | 0.87 | 0 |  |
| Group | 0.63 | 0.43 | 0.01 |  | 1.50 | 0.23 | 0.03 |  |
| Hemisphere | 2.82 | 0.09^+^ | 0.05 |  | 5.49 | 0.02* | 0.09 |  |
| Hemisphere × Group | 0 | 0.96 | 0 |  | 0.79 | 0.38 | 0.01 |  |
| Preview × Direction | 0.02 | 0.88 | 0 |  | 0.35 | 0.56 | 0.01 |  |
| Direction × Preview × Group | 0.06 | 0.81 | 0 |  | 4.39 | 0.041* | 0.07 |  |
| Direction × Hemisphere | 8.97 | 0.004** | 0.13 |  | 51.65 | <0.001*** | 0.47 |  |
| Direction × Hemisphere × Group | 2.02 | 0.16 | 0.03 |  | 1.41 | 0.24 | 0.02 |  |
| Hemisphere × Preview | 1.56 | 0.22 | 0.03 |  | 0.02 | 0.53 | 0.01 |  |
| Hemisphere × Preview × Group | 3.13 | 0.082^+^ | 0.05 |  | 0.03 | 0.86 | 0 |  |
| Direction × Hemisphere × Preview | 0.08 | 0.78 | 0 |  | 2.25 | 0.14 | 0.04 |  |

^+^ *p* < .1. * *p* < .05. ** *p* < .01. *** *p* < .001.
